# From Sourcing to End‐of‐Life: Sustainability Challenges and Opportunities in Organic Bioelectronics

**DOI:** 10.1002/advs.77121

**Published:** 2026-08-17

**Authors:** Gwennaël Dufil, Marine Batista, Andrea Bionda, Erica Zeglio

**Affiliations:** ^1^ Department of Chemistry Wallenberg Initiative Materials Science for Sustainability Stockholm University Stockholm Sweden; ^2^ Department of Neuroscience Karolinska Institutet AIMES – Center for the Advancement of Integrated Medical and Engineering Sciences Solna Stockholm Sweden; ^3^ Department of Chemistry Stockholm University Stockholm Sweden; ^4^ Stockholm University Center for Circular and Sustainable Systems (SUCCeSS) Stockholm University Stockholm Sweden

**Keywords:** device fabrication, end‐of‐life, green chemistry, organic bioelectronics, organic electronic devices, sustainability

## Abstract

The field of organic bioelectronics carries tremendous expectations, driven by the unique possibilities offered by conjugated polymers. These materials promise high‐resolution biological signal transduction, seamless integration with living tissues, ease of processing, and virtually unlimited opportunities for molecular design. Yet, at a time of growing ecological crisis, technological advances must be accompanied by critical reflection on material sourcing, use, waste generation, and mitigation strategies. This perspective addresses sustainability across the full lifecycle of organic bioelectronic materials and devices, providing a foundation for researchers seeking more sustainable alternatives in their work. It examines the sourcing of conjugated polymers, weighing the benefits and limitations of current synthetic routes, greener alternatives, solvents, and monomers in light of sustainability considerations. Moving to device fabrication, it assesses current practices and identifies opportunities for less energy‐ and solvent‐intensive, scalable, and more material‐efficient processes. It further considers present and emerging applications where organic bioelectronics has the potential to provide a positive contribution to sustainability and emphasizes biosafety as well as regulatory challenges they face as the field expands. Finally, device end‐of‐life scenarios are discussed, surveying degradable materials and systems and proposing clearer definitions and standards to align terminology and guide the community toward a shared direction.

## Graphical abstract

Organic bioelectronics bridges biology and electronics using organic mixed ionic/electronic conductors. This Perspective addresses sustainability across the full lifecycle of organic bioelectronic materials and devices, offering a framework to guide material sourcing, synthesis, fabrication, application, and end‐of‐life strategies toward bioelectronic technologies that integrate performance and long‐term sustainability.

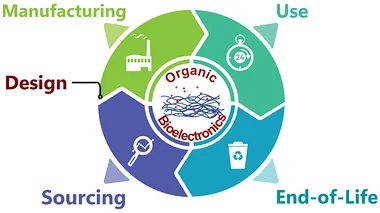

## 1 Introduction

**FIGURE 1:**
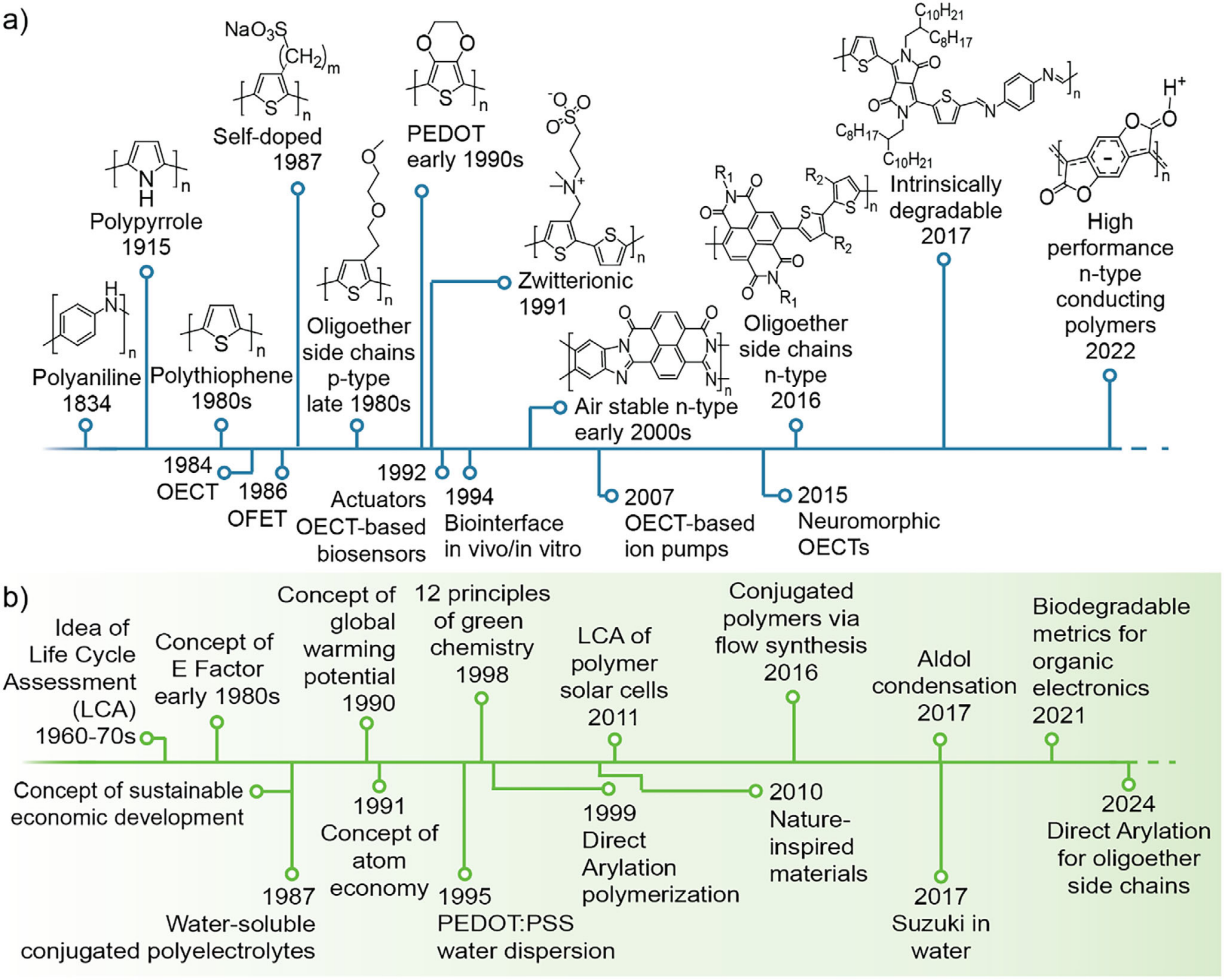
Timelines connecting key developments in organic bioelectronics and sustainability. (a) Timeline of the discovery of the main classes of conjugated polymers for organic (bio)electronics and key milestones in the development of organic bioelectronics devices and bio‐interfaces [3, 4, 5, 6, 8, 9, 10, 15, 16, 17, 18, 19, 20, 21, 22, 23, 24]. (b) Timeline highlighting the emergence of sustainability metrics and the integration of sustainability considerations in organic bioelectronics, including solvent selection, synthetic strategies, nature‐inspired materials, and degradation [25, 26, 27, 28, 29, 30, 31, 32, 33, 34].

Organic bioelectronics was first defined in 2007 as a field centered on carbon‐based devices whose core components, predominantly conjugated polymers, are used to interface with biological specimens [1]. The field builds on initial studies on conjugated polymers, such as polyaniline in 1834 by Runge [2], and polypyrrole in 1915 by Angeli (Figure 1a) [2], culminating in the 1977 discovery by Shirakawa, Heeger, and MacDiarmid that exposing conjugated polymers, such as polyacetylene, to oxidative vapors removes electrons from the polymer backbone and creates free electronic carriers, enabling electrical conduction [3]. This was followed by the discovery of crystallinity in conducting polythiophenes by Garnier et al. in 1985, and subsequently by the synthesis of PEDOT in 1988 by Jonas and Schrader, which has since become one of the most emblematic materials in the field. (Figure 1a) [4, 5, 6]. The redox properties of conducting polymers, which enable them to switch between insulating, semiconducting, and conducting states, gave rise to a new paradigm where electronic and ionic conduction are intimately interconnected [7]. This led to an early demonstration of an organic electrochemical transistor (OECT) by White et al. in 1984 [8], in which a potential applied through an electrolyte modulates the current flowing within a conjugated polymer channel. The study was followed in 1986 by the work of Tsumura et al., in which the potential was instead applied through a dielectric layer, giving rise to what is referred to as an organic field‐effect transistor (OFET) (Figure 1a) [9]. Later, in 1992, Pei and Inganäs demonstrated the actuation behavior of polypyrrole‐based conducting polymers [10]. Today, the types of materials used in organic bioelectronics have expanded beyond conjugated polymers to include small molecules and composites, broadly defined as organic mixed ionic–electronic conductors [11]. Their chemical tunability and mechanical compatibility with biological tissues make them ideal for applications ranging from sensing to stimulation and drug delivery [12, 13]. Their optical absorption and emission across a broad spectral range enable real‐time, non‐invasive monitoring of biological processes through absorbance or fluorescence‐based readouts. Furthermore, their ability to transduce light into electrical or thermal signals enables applications in light‐based stimulation, such as optogenetics or photothermal therapy [14].

Since the early discovery of homopolymers, such as polyaniline and polypyrrole, featuring only one monomer type in their backbones, the chemistry of conjugated polymers has been tailored at the molecular level to provide specific functionalities or improve their performance for applications (e.g., conductivity, stability, and solubility), enabling a wide range of bioelectronic devices (Figure 1). The processing of conjugated polymers from solution enables large‐scale coating and printing, dropping the production cost of organic bioelectronic devices (e.g., a few euros/device for paper‐based OECTs [35, 36]. Importantly, their biocompatibility across several levels of biology [13], and integration into soft architectures (e.g., hydrogels) make them well‐suited for integration with soft tissues, enabling stable and minimally disruptive interfaces with living systems [37]. Conjugated polymers' inherent flexibility also facilitates the development of flexible devices for applications such as wearable or implantable bioelectronic interfaces, where biocompatibility pairs with conformability.

While the field has long looked at structural modifications and processing parameters that provide the best possible performance for applications, sustainability has long been overlooked. Building blocks for conjugated polymers typically originate from the oil industry, and the embodied energy required to produce commercial formulations such as poly(3,4‐ethylenedioxythiophene):polystyrene sulfonate (PEDOT:PSS) is comparable to that of electronic‐grade silicon fabricated by chemical vapor deposition (CVD): i.e., 1.12 GJ kg^−^^1^ and 1 GJ kg^−^^1^, respectively [38, 39]. Indeed, the word “plastic bioelectronics” was introduced as a synonym to refer to organic bioelectronics. Thus, the rise of organic bioelectronics brings the potential risk of generating a new waste stream; while plastics are known for their persistence over time, it is essential to proactively address the environmental impact of these emerging materials and develop sustainable solutions [40].

The three pillars of sustainable development (social, economic, and environmental) were first introduced in 1987 by the economist Edouard Barbier (Figure 1b), who defined it as the “development mode that fulfills the needs of [the] present generation without compromising the ability of future generations to fulfill theirs” [41]. Concepts such as circularity, embodied in the 5Rs principle of Reuse, Repair, Remanufacture, Reprocess, or Recycle, emerged to replace established linear models [42], in which produced materials and goods will ultimately become waste [43]. The cradle‐to‐cradle approach englobes circularity at a larger scale: new compounds that are synthesized should *in fine* return to biological cycles [44]. Following the cradle‐to‐cradle reasoning, researchers and industries develop new products taking into account their end‐of‐life scenarios; avoiding the creation of long‐term waste, such as the recent example of polyfluoroalkyl substances (PFAS) [45]. Both sustainability science and organic electronics emerged during the late 20th century and have since evolved in parallel as distinct scientific disciplines (Figure 1). Concepts such as the 12 principles of green chemistry edited in 1998 by Paul Anastas and John Warner established the first links between these fields (Figure 1b) [25], progressively guiding the development of greener organic bioelectronics. Today, high‐performance materials can be synthesized using a variety of synthetic pathways (as further described in Section 2), while sustainability concepts are increasingly being integrated in materials and device designs. Connecting the emerging field of organic bioelectronics to sustainable practices requires the use of tools and metrics to guide laboratory procedures, particularly regarding the synthetic efficiency of reactions, as captured by metrics such as atom economy and E‐factor, and materials integration into device architectures.

Decision‐making at a broader level requires metrics that extend beyond the laboratory, encompassing material sourcing, device manufacturing, operational lifetime, and end‐of‐life scenarios. Life cycle assessment (LCA) emerged in the 1960s (Figure 1b) [26] to address this need and quantify the environmental impact of materials and devices by accounting for all energy and material flows throughout their production and use. Impacts may arise from the choice of starting materials with a high environmental footprint (material flows) or from the processes used to transform them (process flows). However, their overall contribution will also strongly depend on the duration of the use phase, as well as the end‐of‐life management and disposal pathways of the materials [27]. In this context, commonly used metrics include global warming potential (GWP100), a metric invented in 1990 (Figure 1b) [46], that is expressed in kg CO_2_‐equivalent, and environmental toxicity indicators (freshwater, terrestrial, and human toxicity, including carcinogenic effects), typically reported as 1,4‐dichlorobenzene equivalents (1,4‐DCBe) [47]. While one metric alone might not be enough to evaluate sustainability, combining these metrics in an overall assessment can help the decision‐making process by compensating the downsides of one method with another one.

The guiding question of this perspective is: what are the current practices in organic bioelectronics and how can we move towards more sustainable solutions for the next generation of organic bioelectronic products? To address this, we touch on four different aspects of the value chain: sourcing (Section 2), manufacturing (Section 3), use (Section 4), and end‐of‐life (Section 5). By sourcing, we refer to the origin of materials building blocks and how they are synthesized; manufacturing includes the processes used for materials processing and device fabrication; use relates to the target application, associated performance metrics, and the expected product lifetime; finally, end‐of‐life considers the implementation of degradable materials. For each of these sections, we compile metrics and figures of merit to guide readers in the selection of materials and methods. We also discuss the definitions of key terms, including biodegradable, bioresorbable, dissolvable, biocompatible, and recyclable, clarifying their definition, what the available evidence supports, and where critical knowledge gaps remain. Finally, we discuss strategies for the development of sustainable devices and explore how such devices can contribute to a more sustainable society.

## 2 Conjugated Polymers for Bioelectronics: Structure, Properties, and Design Criteria

The nature of the π‐conjugated backbone in conjugated polymers determines their charge transport characteristics, classifying them as p‐type, n‐type, or ambipolar materials. P‐type conjugated polymers, such as poly[2‐(3,3′‐bis(2‐(2‐(2‐methoxyethoxy)ethoxy)ethoxy)‐[2,2’‐bithiophen]‐5‐yl)thieno[3,2‐b]thiophene] (p(g2T‐TT)) and poly(3‐hexylthiophene) (P3HT), feature a relatively high Highest Occupied Molecular Orbital (HOMO) that facilitates electron extraction (p‐type doping), increasing the concentration of holes. In contrast, n‐type conjugated polymers, like polybenzodifuranedione (PBFDO), poly(benzimidazobenzophenanthroline) (BBL), and the naphthalenediimide‐bithiophene co‐polymer (p(gNDI‐gT2)), are electron‐poor materials that conduct electrons as charge carriers. For many of these materials, doping is possible within the stability window of water, promoting low‐voltage and stable device operation in aqueous environments [48].

While p‐type materials dominate organic bioelectronics due to their comparatively higher ambient stability, the development of n‐type conjugated polymers has been slowed down by stability limitations until the emergence of the first air‐stable n‐type conjugated polymers in the early 2000s, with recent advances now reaching conductivities of up to 2000 S cm^−^^1^, as demonstrated by Tang et al. in 2022 (Figure 1a) [15, 18]. The low‐lying LUMO required to increase electron concentration can also facilitate thermodynamically favorable side reactions, such as oxygen and water reduction, which undermine device stability and efficiency [49]. Overcoming these issues requires molecular designs with sufficiently deep‐lying LUMO levels (below −4.0 eV) that mitigate parasitic effects and preserve the stability of n‐type conjugated polymers [50, 51]. Ambipolar conjugated polymers demand a distinct energetic structure, combining high HOMO and low LUMO, to provide a low band gap (≤1 eV) and to balance both hole and electron mobility. A typical approach involves the co‐polymerization of strong electron‐donating (D) units with strong electron‐accepting ones (A), requiring synthesis routes that provide a high level of structural control [50].

Key energetic properties of conjugated polymers, such as optical bandgap, and charge carriers’ mobility, are affected by several molecular design parameters: degree of planarity, molecular weight, and torsional disorder along the chain, which affect how molecular chains pack both in solution and solid state. In solution, substituents on the backbone play a crucial role to determine polymers solubility. Alkyl and oligo(ethylene glycol) side chains were introduced in the late 1980s by Sato et al. and Bryce et al., respectively, to improve the solubility of p‐type conjugated polymers in organic solvents; comparable advances for n‐type conjugated polymers emerged only in 2016 through the work of Giovannitti et al. (Figure 1a) [17, 21, 22, 50].

In contrast, side chains bearing charged groups, demonstrated by the zwitterionic thiophene polymer reported by Andersson et al. in 1991 [52], impart water solubility to conjugated polymers (Figure 1a) [53, 54]. In thin films, packing of conjugated polymers plays a critical role in key properties, such as charge carrier mobility (μ): the rate at which charge carriers respond to an applied potential (V) [50, 53, 54]. Additionally, polar non‐ionic side chains (e.g., oligo(ethylene glycol)) have been used to combine film stability and hydrated ion penetration in contact with aqueous electrolytes, enabling volumetric capacitance (C*), which quantifies the material's ability to store charge throughout its volume [50, 53, 54, 55, 56]. Although significant progress has been made to improve the properties of conjugated polymers and long‐term device stability for applications, significant concerns remain regarding their toxicity and environmental impact. These issues primarily arise from the synthesis and processing of donor‐acceptor co‐polymers, which often involve resource‐intensive methods and the use of chlorinated solvents. In the following section, we focus on the recent advances in synthetic strategies towards more sustainable conjugated polymers for bioelectronics [57], particularly in the context of OECTs [55, 56].

### 2.1 Sustainable Routes to Conjugated Polymers

**FIGURE 2:**
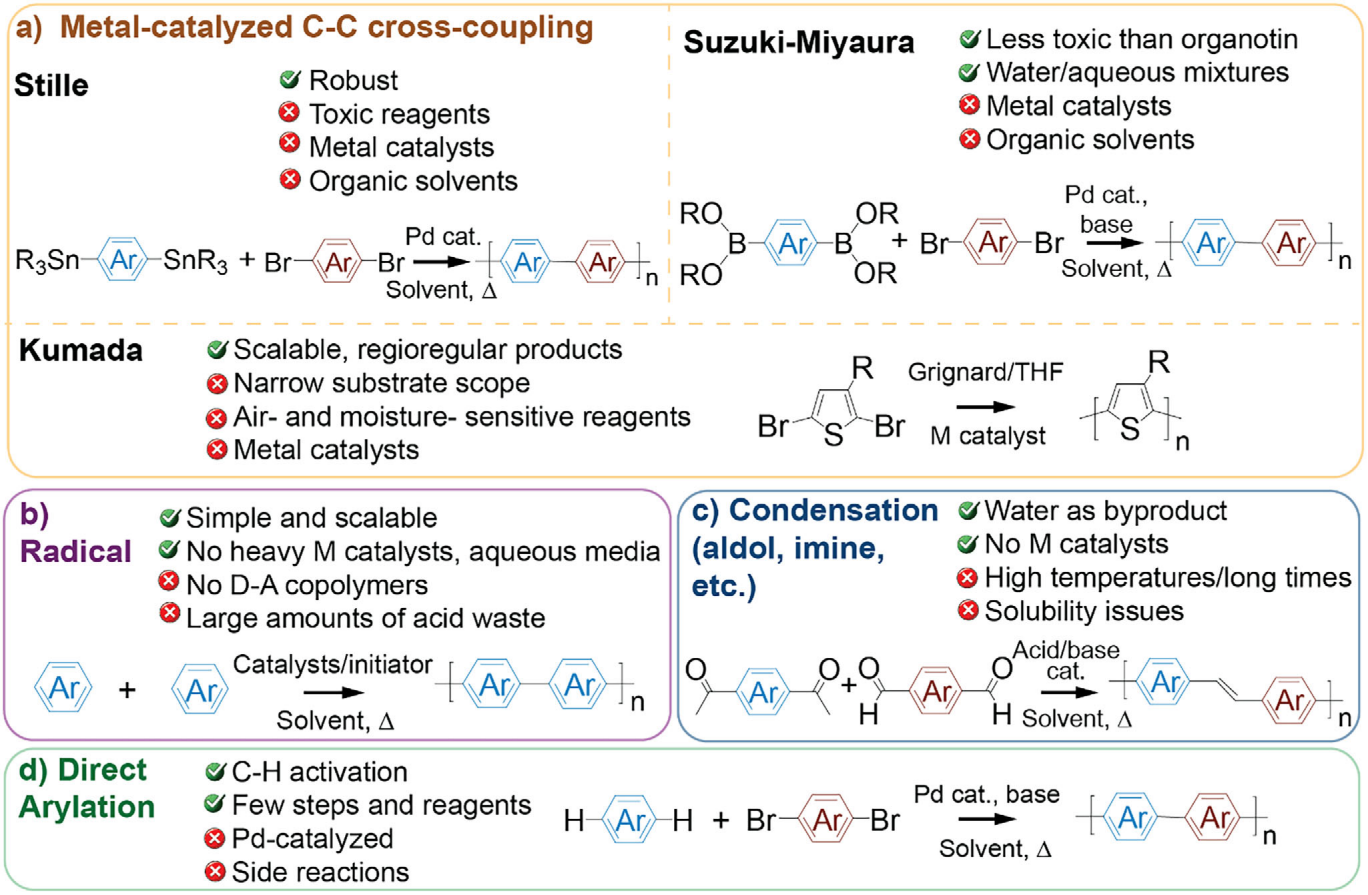
Chemists’ toolbox for the synthesis of conjugated polymers. The main polymerization strategies are listed together with a conceptual illustration of their reaction conditions: (a) Metal‐catalyzed C–C crosscoupling (e.g., Stille, Suzuki‐Miyaura, and Kumada), radical polymerization, condensation (e.g., aldol condensation), and direct arylation polymerization (DArP). Each reaction type is accompanied by its main advantages and limitations in terms of both sustainability and performance, and representative reaction schemes highlighting the characteristic transformations, bond formations, and species involved.

Most donor‐acceptor conjugated polymers are synthesized using transition metal‐catalyzed coupling reactions, such as Stille, Suzuki‐Miyaura, and Kumada coupling (Figure 2 and Table 1).

**TABLE 1:** Comparison of classic and sustainable methods used to synthesize conjugated polymers for bioelectronics.

Reaction type	Advantages	Disadvantages	Type of species involved
Stille coupling	Robust for many D‐A backbones; tolerant to a wide range of functional groups; Low defect density in the final polymer.	Uses toxic organostannane reagents; Requires expensive Pd catalyst; Hazardous organic solvents and waste; Metal and tin recovery/recycling is difficult.	Monomers: Organostannanes and aryl bromides (or other aryl halides); Catalyst: Pd catalyst; Solvents: Aromatic hydrocarbons (e.g., toluene, xylene), ethers (e.g., THF and 1,4‐dioxane) or polar aprotic solvents (e.g., DMF and DMSO).
Suzuki‐Miyaura coupling	Less hazardous than Stille; Broad functional‐group tolerance; Demonstrated scalability [57].	Less efficient/robust than Stille; Homocoupling side reactions can occur; Requires Pd catalyst, base, and significant solvent volume.	Monomers: Boronic acid or ester and aryl halide; Catalyst: Pd catalyst; Base: Inorganic or organic; Solvents: Aromatic hydrocarbons, ethers, or polar aprotic solvents. Some protocols report water as pure solvent or cosolvent.
Kumada	Effective for regioregular polythiophenes; Enables chain‑growth with good control over molecular weight and dispersity; Uses inexpensive Grignard reagents; Well suited to scale‑up and, in some cases, continuous/flow processing	Grignard reagents are air‑ and moisture‑sensitive; Relies on Ni or Pd catalysts, with associated metal footprint; Generates Mg salt and halide waste; Functional‑group tolerance is more limited than in Suzuki or Stille couplings.	Monomers: Aryl halides and Grignard reagents (e.g., halo‑thiophenes and corresponding Mg derivatives); Catalyst: Ni or Pd complex, often with phosphine ligands Solvents: Ethereal solvents (e.g., THF, diethyl ether) and/or aromatic hydrocarbons (e.g., toluene), under inert atmosphere.
Radical polymerization	Simple and scalable; Metal‐free or uses relatively benign oxidants (e.g., FeCl_3_); Can enable in‐situ film formation and in‐situ doping; Often compatible with aqueous or mixed aqueous media.	Limited control over molecular weight or regioregularity; Poor solubility and processability are common; Residual oxidant and by‐products can remain in the material; Narrow monomer scope; donor‐acceptor architectures are difficult to access.	Monomers: unsaturated or heteroatomic species that can generate radical intermediates (e.g., aniline, pyrrole, thiophene, EDOT) Initiator/oxidant: FeCl_3_ or other oxidizing/reducing agents; Solvents: THF, DMF, DMSO, toluene, alcohols, or water‐alcohol mixtures. Sometimes chlorinated solvents.
Aldol condensation	Water is the only by‐product; Metal‐free; Structural versatility (rigid and fused systems possible); Broad monomer scope.	Less explored compared to Pd‐catalyzed routes; Often requires elevated temperature or strong acid/base.	Monomers: A carbonyl compound and one bearing an α‐hydrogen adjacent to a carbonyl; Catalyst: Acid or base (e.g., p‐toluenesulfonic acid or NaOH) Solvents: Toluene, polar aprotic solvents, or water/alcohol mixtures.
Direct arylation polymerization (DArP)	Direct C‐H bond activation (no pre‐functionalization steps); Fewer synthetic steps than cross‐coupling routes; Reduced use of hazardous reagents and improved atom economy.	Less versatile and predictable than Stille; Requires extensive reaction optimization; Homocouplig and β‐defects can occur, affecting materials performance.	Monomers: An aryl halide and an aryl monomer Catalyst: Pd or Cu catalyst with ligands; Additives or co‐catalysts: Inorganic bases and carboxylates; Solvents: polar aprotic (DMF) or greener solvents (e.g., DMAc, water, ethanol, water/alcohol mixtures)

Metal‐catalyzed cross‐coupling methods have been introduced to enable the precise synthesis of donor–acceptor (D‐A) and regioregular conjugated polymer structures (Figure 2a and Table 1). The main drawbacks of these reactions are the need for heavy metal catalysts (often palladium) and a halide‐functionalized monomer (often bromide), introducing an extra functionalization step with respect to reactions that do not require prior functionalization.

Among these methods, the Stille reaction is well‐established, robust, tolerant towards various monomer functionalities, and it typically produces polymers with low defect levels, including minimal homocoupling and positional defects. However, a key limitation is the need for toxic organostannane monomers. Suzuki–Miyaura is generally less robust than Stille, with homocoupling as the primary side reaction [58]. Homocoupling defects are detrimental to the final polymer properties because the regular D‐A sequence is altered with D‐D and A‐A units. These perturb the electronic structure and backbone conjugation, impairing charge transport, reducing carrier mobility, and compromising the overall functional performance of the thin film. However, Suzuki‐Miyaura involves less hazardous boronic acids or esters instead of organostannane monomers, making it more suitable for large‐scale production. Kumada coupling employs Grignard reagents and is attractive due to its low‐cost catalysts and high reactivity, but its poor functional group tolerance and stringent moisture and air sensitivity limit its applicability for complex monomer systems [59].

All these reactions rely on metal catalysts, demanding solvent‐ and energy‐intensive purification steps. Kosko et al. showed that Soxhlet extraction of 400 mg of poly(9,9‐dioctylfluorene‐alt‐benzothiadiazole) (F8BT) nanoparticles using methanol, acetone, and n‐hexane as solvents, led to over 1000 ppm (0.1 wt.%) of Pd residue inside the polymer. Further purification using gel permeation chromatography (GPC) led to 195 ppm of Pd. Only after two additional washes with sodium diethyldithiocarbamate, the Pd concentration was found below 1 ppm. Such metal residues have been shown to negatively impact charge carrier mobility of conjugated polymers. Griggs et al. showed that preparative GPC purification led to a 92% increase in OECT electron mobility for p(C6g3NDI‐T), and ∼50% increase in hole mobility for p(g4T2‐TT) relative to the unpurified polymers. These differences are attributed to a substantial decrease in Pd levels from 1769 to 39 ppm for the p‐type polymer and from 1176 to 73 ppm for the n‐type polymer.

These residual metal catalysts and byproducts from the synthesis, in addition to a negative impact on device performance (up to 60% reported performance loss for the p‐type p(g4T2‐TT) and 80% loss for the n‐type p(C6g3NDI‐T)) [60], can pose significant regulatory and safety barriers, particularly for medical devices that must comply with stringent biocompatibility and toxicology standards (as discussed in Section 5) [58]. Consequently, there is a growing need to adopt more sustainable synthetic strategies that phase out toxic precursors/catalysts or enable their complete removal andrecycing post‐synthesis, and improve the overall environmental profile of next‐generation bioelectronic materials.

Radical polymerization provides a more sustainable approach with respect to cross‐coupling methods by avoiding toxic transition metals and monomer functionalization. The polymerization proceeds through reactive radical intermediates, typically radical cations or radical anions, generated under electrochemical or chemical oxidation/reduction conditions. Key advantages include simplicity, transition‐metal‐free conditions (reducing metal contamination and costs), the possibility of using water as a solvent [15],  and good scalability. Important conjugated polymers synthesized via this method include polypyrrole, polythiophene, and poly(3,4‐ethylenedioxythiophene). Furthermore, incorporating charge‐balancing counterions in situ enables the direct formation of conductive polymers with high conductivity and electrochemical activity immediately upon synthesis, eliminating the need for post‐synthetic doping steps [60, 61]. Key materials synthesized via this method include poly(3,4‐ethylenedioxythiophene):poly(styrenesulfonate) (PEDOT:PSS), obtained by oxidative polymerization of EDOT with persulfate/iron(III) salts in an aqueous PSS solution, and polybenzodifuranedione (PBFDO), via oxidative polymerization of 3,7‐dihydrobenzo[1,2‐b:4,5‐b′]difuran‐2,6‐dione (HBFDO) using duroquinone (TMQ), yielding an n‐doped, highly conductive polymer directly from synthesis [15, 62]. Radical polymerization can be initiated by chemical oxidants (oxidative polymerization), light (photopolymerization) [13], enzymes (enzymatic polymerization) [63, 64], or electrical currents (electropolymerization).

The absence of toxic reagents during the synthesis opens up to the direct use of polymers without the need for purification. Huang et al. demonstrated this approach for PBFDO, where substituting TMQ with α‐tocopherylquinone (α‐TQ) suppressed catalyst aggregation during the reaction and eliminated the need for post‐reaction dialysis without decreasing conductivity (1321 ± 67 S cm^−^^1^ for α‐TQ‐synthesized without dialysis vs. 1351 ± 85 S cm^−^^1^ for TMQ‐synthesized with dialysis) [65]. However, it´s important to note that this approach lacks the precise control needed for synthesizing donor‐acceptor copolymers and offers poor control over polymer regioregularity compared with cross‐coupling methods [28, 29, 30].

Condensation methods stand out as more sustainable alternatives to metal‐catalyzed reactions for the synthesis of D‐A copolymers (Figure 2c). Key advantages are that water is the only by‐product and it eliminates reliance on toxic metal catalysts [66].

Aldol polycondensation enables unique rigid‐rod backbones with remarkable structural versatility, allowing the synthesis of both half‐fused and fully fused conjugated polymers with D‐A or acceptor–acceptor (A‐A) architectures and tunable electronic properties. Zhang et al., in their seminal work published in 2017 (Figure 1b) [33], showed that this method can provide a greener alternative to conventional Stille polymerization for isoindigo‐based conjugated polymers such as PIID‐DT and PBIBDF‐DT, avoiding toxic organotin reagents and producing polymers with high molecular weights (M^GPC^_n_ up to 53.9 kDa). However, this route led to lower OFET mobilities up to 0.16 cm^2^ V^−^^1^ s^−^^1^ for PIID‐DT (p‐type) and 0.26 cm^2^ V^−^^1^ s^−^^1^ for PBIBDF‐DT (n‐type) with respect to their Stille‐prepared counterparts (Table 2). Wu et al. used electrospray deposition and scanning tunnelling microscopy (ESD‐STM) to show that n‐type co‐polymers prepared via Aldol condensation suffer from sequence defects, resulting from the wrong ordering of comonomers, and coupling defects, due to incorrect coupling position or orientation of comonomers [67]. Another drawback of Aldol polycondensation is the restricted monomer scope, requiring at least one component bearing an active methylene (CH_2_) or methine group adjacent to a carbonyl, and a complementary aldehyde or ketone. This narrows the range of accessible D‐A combinations compared with cross‐coupling, where almost any halide/organometal pair can in principle be coupled [68].

**TABLE 2:** Conjugated polymers synthesized via conventional and more sustainable methods. This summary identifies conjugated polymers synthesized via conventional and more sustainable routes and compares polymerization method, catalyst type, polymerization solvent, purification solvent (all polymers listed were purified via Soxhlet extraction followed by re‐precipitation), simple E‐Factor, molecular weight, and reported best charge‐carrier mobilities across polymerization routes. Metrics are only stated for the polymerization step and don´t account for monomer preparation.

Material	Type	Polymerization method/catalyst	Polymerization solvent	Purification solvent	Simple E‐factor	Mn/Mw (kDa)	Mobility (cm^2^ V^−1^ s^−1^)	Reference
PIID‐DT 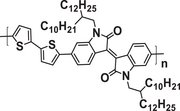	p	Aldol/pTsOH	Toluene	Methanol, hexane, dichloromethane and chloroform	—	59.5/131	0.16 ^a^, ^c^	[33]
	p	Stille/Pd_2_(dba)_3_	Toluene	Methanol, hexane, chloroform	—	20.2/64.5	0.25 ^a^, ^c^	[33]
PBIBDF‐DT 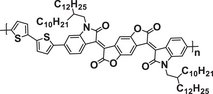	n	Aldol/pTsOH	Toluene	Methanol, hexane, dichloromethane and chloroform	—	31.9/73.6	0.26 ^a^, ^c^	[33]
	n	Stille/Pd_2_(dba)_3_	Toluene	Methanol, hexane, chloroform	—	29.8/44.7	0.62 ^a^, ^c^	[33]
PNDIT2 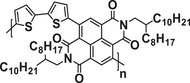	n	DArP/Pd_2_(dba)_3_	Toluene	Acetone, ethyl acetate, and hexanes	—	31/91	2.9 ^a^	[69]
	n	Stille/Pd_2_(dba)_3_	Toluene	Methanol, acetone, hexane and chloroform	—	32/174	3.2 ^a^	[69]
PTDPP‐F4 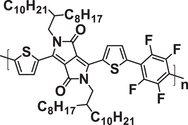	n	DArP [70]/Pd(OAc)_2_	Toluene/DMAc	Methanol, ethyl acetate, chloroform	—	14.0/51.8	3.0 ^a^, ^c^,^c^[71]	[70, 72]
	n	Stille/Pd_2_(dba)_3_	Toluene/DMF	Methanol, acetone, ethyl acetate, hexane and chloroform	—	16.3/29.2	2.4 ^a^, ^c^	[73]
	p	Stille/Pd_2_(dba)_3_			—	16.3/29.2	0.25 ^a^, ^c^	[73]
IDT‐BT 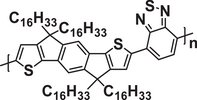	p	DArP/Pd_2_(dba)_3_	Mesitylene	Methanol, acetone, ethyl acetate, n‐hexane, chloroform	—	79/214	2.4 ^a^	[74]
	p	Suzuki/Pd_2_(dba)_3_	Toluene/water 5:1	Methanol, acetone, hexanes	—	123/322	2.8 ^a^	[74]
	p	Suzuki/Pd_2_(dba)_3_	Toluene/water 5:1	Methanol, acetone, hexanes	—	61/117	1.2 ^a^	[74]
p(g3TT‐T2) 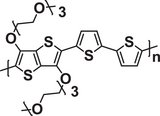	p	DArP/Pd_2_(dba)_3_	Toluene	Hexane, methanol, ethyl acetate, chloroform	48	29/63.8	2.0 ^b^	[75]
	p	ADAP/Pd(OAc)_2_	NBP	Hexane, ethyl acetate, chloroform	17	20/40	6.1 ^b^	[28]
p(g3T2‐TT) 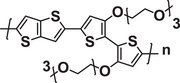	p	Stille/Pd_2_(dba)_3_	NMP	Hexane, diethyl ether, acetone, methanol, chloroform	60	10/20	0.95 ^b^	[55]
P3HT 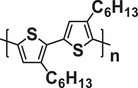	p	DArP/Pd(Herrmann‐Beller)	THF	Acetone and hexanes	—	33/59,4	0,19^a^, ^c^	[71, 76]
	p	Rieke/Zn and Ni(dppe)Cl_2_	THF	Methanol, HCl, chloroform	—	25/32,5	0,02 ^a^, ^c^	[71]
	p	GRIM/MgBr_2_ and Ni(dppp)Cl_2_	THF	Methanol, hexanes, and chloroform	—	88/132	0,11 ^a^, ^c^	[71]

^a^ OFET;

^b^ OECT;

^c^ after annealing

Direct arylation polymerization (DArP) was introduced in 1999 by Sevignon et al. (Figure 1b) as a more sustainable method for the synthesis of D‐A co‐polymers and, in 2024, was used by Kimpel et al. to syntesized oligoether conjugated polymers [28, 32]. By exploiting C─H bond activation, DArP bypasses the preparation of organometallic monomers, thereby eliminating at least one to two synthetic steps per monomer and avoiding the use of pyrophoric organolithium reagents or toxic stannane/borane intermediates (Figure 2d) [28, 77, 78, 79, 80, 81]. Emulsion‐based DArP variants have demonstrated up to a 10‐fold reduction in organic solvent use compared with conventional Stille polymerization [82].

However, DArP requires resource‐ and time‐consuming optimization of reaction conditions to suppress side reactions such as homocoupling and β‐defects, which can arise from unselective activation of multiple C─H bonds. DArP also typically yields lower molecular weight polymers compared to Stille or Suzuki coupling, which can negatively impact device performance [83].

While synthetic routes are commonly optimized to minimize the number of steps and solvent use, quantitative metrics are essential to objectively assess and compare the true environmental footprint of different polymerization strategies. In 2017, Sheldon provided a comprehensive overview of quantitative green chemistry metrics, including atom economy, the E‐factor, and LCA, and discussed their application to evaluate the sustainability of chemical processes, primarily in the context of pharmaceuticals and fine chemicals [84]. The idea of E‐factor emerged in the early 1980s [30]. It evaluates efficiency by quantifying the total mass of waste generated per unit mass of product, and it can be divided into two types: simple E‐Factor, measuring the waste from the chemical synthesis alone, and complete E‐factor, accounting for the entire multi‐step synthetic pathway, including considerations of water and organic solvents used during the purification step. Atom economy (mol. wt. of product/sum of mol. wts. of starting materials) is a sustainable metric introduced in 1991 by Trost that assesses how efficiently a reaction converts reactants into the desired product [31]. Energy‐efficient conditions and choice of reagents, such as reactions at room temperature, under aerobic conditions, with non‐hazardous/toxic catalysts/building blocks, and employing benign solvents, are equally critical to reduce the overall environmental footprint of conjugated polymer synthesis [85, 86]. These are captured in part by the Reaction Mass Efficiency (RME), which is the percentage of total mass of desired product divided by the total mass of the reactants used, and by the synthetic complexity index (SC Index), which summarizes the number of synthetic steps, overall yield, workup procedures, hazards, and raw material costs associated with a given route, as well as by metrics for energy consumption and solvent sustainability scores [87].

To integrate these factors, Osorio‐Tejada et al. compiled 12 green chemistry metrics into an LCA endpoint measurement to support decision‐making in C─C coupling methods such as Heck and Suzuki polymerizations [88]. The authors compared the environmental impacts of solvents, catalysts, and halobenzene precursors against synthetic performance to guide process selection, finding that solvent selection is the primary driver of environmental impact in C─C cross‐coupling reactions, and that LCA‐based evaluation based on multiple metrics, rather than reliance on a single green chemistry metric, is necessary to identify truly sustainable reaction conditions, as no single protocol performs optimally across all environmental impact categories.

To date, green metrics have been applied sporadically to conjugated polymer synthesis for bioelectronics (Table 1), and no equivalent multi‐metric sustainability analysis has been performed, representing a critical gap to be addressed to guide the rational selection of greener polymerization strategies in this field.

Li et al. developed the water‐soluble oxidant sodium 3‐(2,4,5‐trimethyl‐3,6‐dioxocyclohexa‐1,4‐dien‐1‐yl)propanoate (TMQ‐PANa) to enable the synthesis of poly[(2,2′‐(2,5‐dihydroxy‐1,4‐phenylene)diacetic acid)‐stat‐3,7‐dihydrobenzo[1,2‐b:4,5‐b′]difuran‐2,6‐dione] (PDADF) in water. The Authors showed that TMQ‐PANa precipitates as the water‐insoluble 6‐hydroxy‐5,7,8‐trimethylchroman‐2‐one (HTMCO), enabling 74% recovery of the oxidant by solvent extraction, leading to an E‐factor more than 40‐fold lower than that of the water‐insoluble analogue PBFDO and other top‐performing organic (semi)conductors [89]. It is, however, important to note that only the polymerization and purification steps were considered, and steps like monomer and catalyst synthesis were not included.

Direct arylation polymerization (DArP) intrinsically offers higher atom economy than conventional C─C cross‐coupling methods because it forms C─C bonds through direct activation of aromatic C─H bonds, eliminating the need for pre‐functionalized monomers [75]. For example, Matsidik et al. reported the synthesis of PNDIT2 via DArP with a 35% reduction in production costs while maintaining charge transport properties comparable to those of the Stille‐derived polymer (2.9 versus 3.2 cm^2^ V^−^^1^ s^−^^1^, respectively) [69]. However, achieving high‐performance materials through DArP remains challenging, as polymer quality is highly sensitive to reaction conditions and catalyst selection. Li et al. synthesized the p‐type polymer IDT‐BT using a Pd_2_(dba)_3_/P(o‐MeOPh)_3_/PivOH/Cs_2_CO_3_/o‐xylene system at 100 °C, yielding a low‐molecular‐weight polymer (Mn = 15 kDa) containing approximately 5% structural defects, which resulted in a modest hole mobility of 0.17 cm^2^ V^−^^1^ s^−^^1^ [90]. Similarly, Broll et al. observed significant homo‐coupling defects during the DArP synthesis of the n‐type polymer PDPPTh2F4, leading to a material with Mn = 30 kg mol^−^^1^ and a maximum electron mobility of 0.60 cm^2^ V^−^^1^ s^−^^1^ [72].

Nevertheless, several pioneering studies demonstrated that DArP can rival, and in some cases surpass, the performance of polymers prepared by classical cross‐coupling methods when reaction conditions are carefully optimized. For instance, Ponder Jr. et al. developed an optimized DArP protocol that yielded defect‐free, high‐molecular‐weight IDT‐BT (Mn = 79 kg mol^−^^1^, Mw = 214 kg mol^−^^1^) with a hole mobility of 2.3 cm^2^ V^−^^1^ s^−^^1^, comparable to that of the corresponding Suzuki‐derived analogue (1.9 cm^2^ V^−^^1^ s^−^^1^) [74]. Furthermore, Luzio et al. demonstrated that combining uniaxial alignment with thermal annealing enabled PDPPTh2F4 to achieve a gate‐bias‐independent electron mobility of 3.0 cm^2^ V^−^^1^ s^−^^1^, outperforming the analogous polymer synthesized via Stille polycondensation (2.4 cm^2^ V^−^^1^ s^−^^1^) [70].

While these works show that DArP can be an effective method to cut down synthetic steps in the synthesis of high‐performance D‐A copolymers, a key question remains about its true impact on sustainability metrics.

Kimpel et al. addressed this aspect by calculating SC Index, normalized energy, reaction mass efficiency, simple E‐Factor, and reaction time for the polymerization of p(g_3_TT‐T2) via DArP and ADAP (Ambient Direct Arylation Polymerization, carried out in air at room temperature) and compared the results with those of p(g3T2‐TT) and p(g4T2‐TT) synthesized via Stille. Their calculations showed that, despite avoiding organotin monomers, DArP of p(g_3_TT‐T2) led to relatively high SCI and reaction mass efficiency of 75 and 79, with respect to 73 and 74 for p(g_3_T2‐TT) synthesized via Stille, respectively.

The Authors used continuous droplet flow synthesis using ADAP and a green solvent (i.e., N‐butyl‐2‐pyrrolidone) to overcome these sustainability limitations, affording p(g_3_TT‐T2) with a considerably increased sustainability score across all four metrics and a similar reaction time compared to DArP (see Table 2) [28, 77, 78, 79, 80]. Moreover, the polymer prepared via ADAP exhibited OECT hole mobilities up to 6.1 cm^2^ V^−^^1^ s^−^^1^, surpassing those obtained with conventional DArP and Stille polymerization (2.0 and 0.95 cm^2^ V^−^^1^ s^−^^1^, respectively).

Under optimized conditions employing a Herrmann–Beller catalyst system, DArP of 2‐bromo‐3‐hexylthiophene affords highly regioregular head‐to‐tail P3HT. As demonstrated by Wang et al. and subsequently by Pouliot et al., this approach provides near‐quantitative yields, number‐average molecular weights of 30.6–33 kDa, dispersities around 1.60, regioregularities ranging from 98 to >99.5%, and minimal structural defects, including suppressed β‐branching and <1% homocoupling defects [71, 76].

Compared with commercially available GRIM (Grignard Metathesis, a type of Kumada polymerization where the AB‐like monomer is generated in‐situ to improve the regioregularity) and Rieke P3HT samples, DArP‐derived polymers exhibited comparable or superior thermal and electronic properties, including a melting temperature of 237 °C and hole mobilities reaching 0.19 cm^2^ V^−^^1^ s^−^^1^, outperforming the Rieke analogue (0.02 cm^2^ V^−^^1^ s^−^^1^) and slightly exceeding GRIM‐derived materials (0.11 cm^2^ V^−^^1^ s^−^^1^) [71]. However, no sustainability metrics were calculated, preventing a true comparison between the sustainability of the three different approaches.

Overall, DArP and ADAP have shown to be suitable alternatives to traditional coupling strategies. Particularly, ADAP offered higher scores in green chemistry metrics and conjugated polymers with comparable device performance to those synthesized via traditional routes. However, its wide scope to other monomer types has yet to be demonstrated [75]. In many cases, the lack of sustainability metrics prevents a true comparison between the different approaches, calling for a more systematic reporting alongside performance metrics. Prioritizing safer, renewable solvents and environmentally benign catalysts is also essential, yet not captured by metrics such as the E‐factor or atom economy. LCA analysis in this matter could help to portray sustainability more accurately.

### 2.2 Solvents Considerations

A critical barrier to the sustainable development of conjugated polymers for bioelectronics is the widespread reliance on toxic, polar aprotic solvents during synthesis and processing. Such solvents pose significant risks due to their toxicity, flammability, environmental persistence, as well as broader impacts such as ozone depletion and contribution to climate change. Common examples include halogenated solvents (e.g., chloroform and carbon tetrachloride), aromatic hydrocarbons (e.g., benzene and toluene), and polar aprotic solvents such as N,N‐dimethylformamide (DMF), N‐methyl pyrrolidone (NMP), and N,N‐dimethylacetamide (DMAc). Although valued for their solubilizing power and ability to stabilize charged intermediates, their use raises serious health and environmental concerns. Regulations such as the European Union's REACH framework impose strict limits on these substances, posing increasing pressure on the chemical and materials science communities to develop safer, effective alternatives for conjugated polymer synthesis [91, 92].

Solvents vary in their greenness and sustainability, as no single option satisfies all environmental, safety, and economic criteria. Instead, a solvent may be considered greener than alternatives for a given application if it is derived from renewable resources, exhibits lower toxicity and volatility, or is produced and managed through more energy‐efficient and waste‐minimizing processes. Thus, solvent selection depends not only on intrinsic properties but also on performance within the entire process, including recovery and recycling, to ensure that improvements in one stage do not introduce greater impacts elsewhere [93].

**FIGURE 3:**
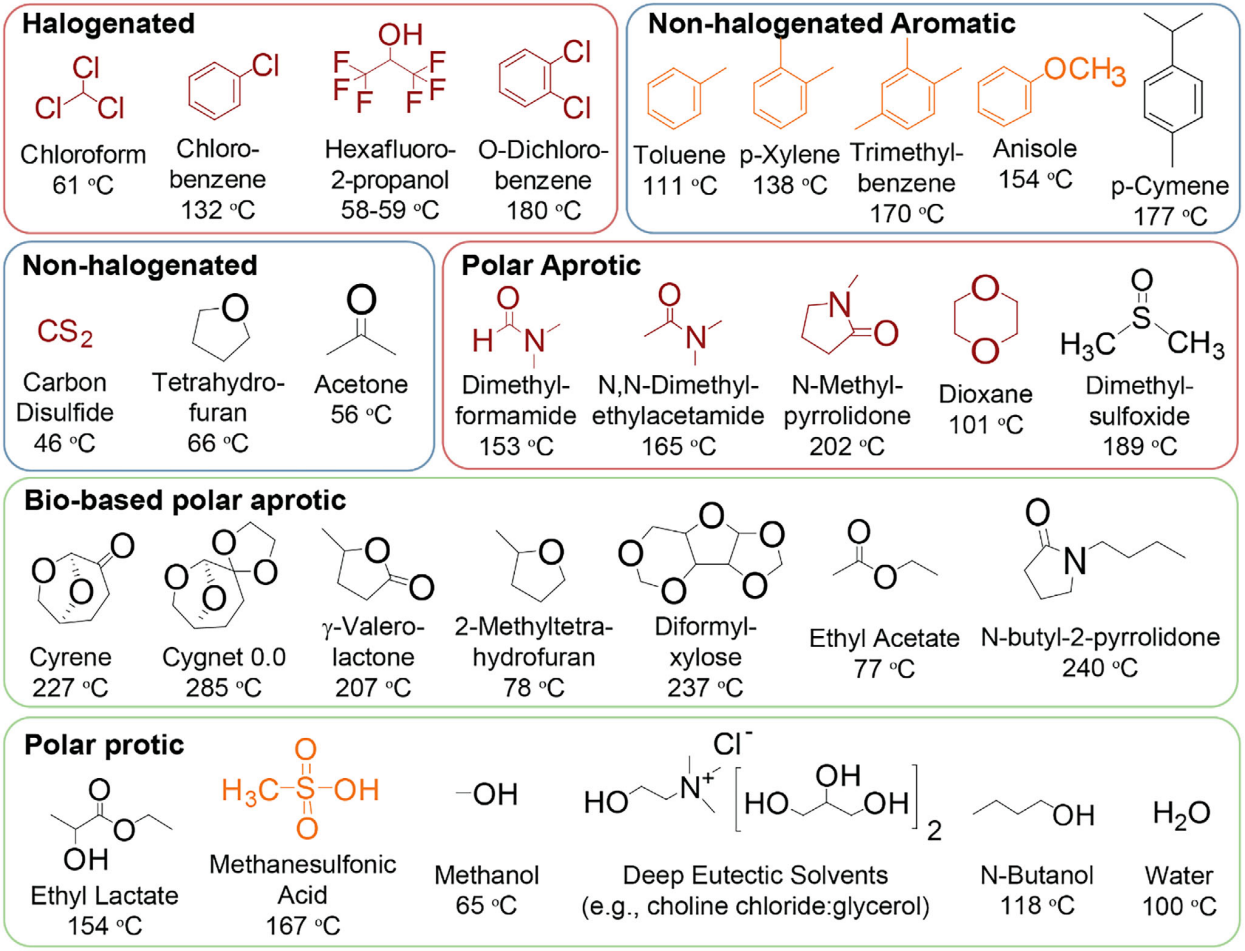
Main types of solvents used in organic bioelectronics. Solvents are classified by chemical nature (halogenated, non‐halogenated aromatic, non‐halogenated, polar aprotic, bio‐based polar aprotic, and polar protic) and hazard level, ranging from red (well‐known toxic solvents) to orange (toxic or corrosive), and black (safer, more sustainable solvents). The corresponding boiling points are reported below each solvent.

Prat et al. introduced a hazard‐driven solvent selection framework for the pharmaceutical and fine‐chemical sectors, extending classical solvent guides to neoteric and bio‐derived solvents through a simple Safety, Health, and Environment ranking into recommended, problematic, or hazardous categories (Figure 3) [94]. Its main value lies in providing a practical, easily updated preliminary assessment based on readily available data, while also highlighting critical data gaps that can limit the industrial adoption of greener solvents.

Major classes of sustainable solvents show promise in conjugated polymer synthesis, though each presents its own benefits and limitations. Cyrene, diformylxylose (DFX), and 2‐methyltetrahydrofuran (2‐MeTHF) have emerged as promising candidates for C─C bond‐forming reactions, including cross‐coupling and polymer synthesis. Cyrene, derived from cellulose, can replace traditional polar aprotic solvents in Suzuki and Sonogashira reactions and has been used in the synthesis of organic materials, offering low toxicity and facile removal, though its stability may be compromised under strongly acidic or basic conditions [95].

DFX, produced from xylose, provides comparable performance to DMF and NMP in Heck cross‐couplings and benefits from its non‐mutagenicity and ease of recovering, but its high viscosity and melting point impose practical limitations [96]. 2‐MeTHF, sourced from biomass, is especially effective in Grignard‐based polymerizations such as P3HT synthesis, improving process safety and throughput, although challenges remain regarding flammability and long‐term feedstock sustainability [97]. Furthermore, Ye et al. reported that the green solvent p‐cymene, a bio‐derived aromatic solvent obtained from citrus‐processing essential oils, can be effectively employed in DArP to synthesize high‐molecular‐weight (Mn up to 51.3 kg mol^−1^), defect‐free conjugated polymers with improved yields and reduced cross‐linking relative to conventional hazardous solvents such as DMA, THF, and toluene [82]. Other candidates, such as γ‐Valerolactone (GVL), offer appealing green metrics but have yet to see widespread application in these transformations [92, 93, 98].

Generally, high‑boiling solvents allow a reaction to be heated more without large evaporation losses, which can be useful for driving slower synthetic steps or improving solubility of high‑molar‑mass conjugated species.

The choice of solvent also plays a critical role in device film formation. Solvents commonly used for conjugated polymer processing generally require high polarizability to match the chemical characteristics of the polymers, often resulting in the use of chlorinated solvents when low boiling points are additionally required. Solvent blending strategies can partially circumvent these limitations by enabling low‐boiling components to evaporate rapidly while retaining the polymer‐solubilizing fraction on the substrate, thereby promoting optimal film rearrangement and morphology formation. Corzo et al. showcased this concept for solar cells, reporting a comparable power conversion efficiency of 15.7% using an eucalyptol/tetralin mixture with respect to 15.9% for chloroform‐based systems [99]. Similarly, Xu et al. demonstrated that blending n‐butanol with DMSO improved the processability of PBFDO compared with pure DMSO, while matching ITO performances as an anode in Organic Light‐Emitting Diodes (OLEDs) [100]. Lastly, Savva et al. showed that the incorporation of 15% acetone into chloroform significantly enhanced ion intercalation within face‐on crystallites, resulting in a threefold increase in transconductance in OECT devices [101]. Beyond the optimization of reaction conditions, novel synthetic methods and processing strategies seek to enhance the sustainability of chemical synthesis through the reduction of synthetic steps, reagent consumption, and solvent use.

### 2.3 Emerging Methods and Techniques for Conjugated Polymer Synthesis

Beyond solvent choice, enhancing synthetic efficiency and selectivity is expected to increase yields while minimizing solvent use and purification requirements.  Emerging techniques such as micellar catalysis, mechanochemistry, and flow chemistry have been introduced to increase process efficiency and sustainability. These approaches, especially when combined with green polymerization methods such as aldol condensation or DArP, offer promising routes toward more sustainable synthesis, as exemplified by the work of Kimpel et al. [28].

Micellar catalysis employs surfactants to enable the synthesis of water‐insoluble conjugated polymers in aqueous media, thereby reducing the need for organic solvents. The process is driven by the hydrophobic effect, wherein hydrophobic monomers and catalysts are concentrated within the micelle cores formed by amphiphilic molecules in water, enhancing reaction rates. This approach has been successfully applied to perform a Suzuki‐Miyaura coupling in water by Mattiello et al. in 2017 (Figure 1b) [102], significantly lowering the use of toxic organic solvents and improving safety. Advantages of micellar catalysis include drastically reduced catalyst loadings (down to ppm levels), mild reaction temperatures, and aerobic conditions, all of which contribute to increased energy efficiency and operational simplicity. Moreover, the micellar environment can stabilize reactive intermediates and enable unique chemo‐ and stereoselectivity. Industrial scalability and compatibility with flow chemistry have also been demonstrated, underscoring its potential for large‐scale production. For example, Sanzone et al. demonstrated that the polycondensation of 9,9‐dioctylfluorene‐2,7‐diboronic acid with dibromo thiophene in an aqueous Kolliphor EL micellar solution under ambient conditions produces polymers with molecular weight and optical properties comparable to those obtained in conventional organic solvents, while reducing the simple E‐factor by an order of magnitude (excluding solvent recovery via distillation) [103]

Despite these advantages, micellar synthesis faces several limitations. Reaction kinetics and polymerization outcomes can be highly sensitive to the choice of surfactant and small differences in reaction conditions, often requiring extensive screening. Limited polymer solubility or precipitation can also occur, sometimes requiring small amounts of organic cosolvents to maintain effective micellar emulsions [103]. Additionally, the environmental fate and degradability of many designer surfactants remain insufficiently understood, demanding further investigation to fully validate the sustainability claims. Yet, the application of micellar‐synthesized materials in organic bioelectronic devices remains to be demonstrated and could offer a promising research direction.

Mechanochemistry offers distinct advantages for organic synthesis, especially for conjugated polymers. Its primary environmental benefit is the drastic reduction, or complete elimination, of solvents, aligning with green chemistry principles and reducing chemical waste, operational costs, and associated hazards. Once milling parameters are optimized (e.g., frequency, ball size/material, and additives), mechanochemical synthesis can proceed under ambient conditions, at room temperature and without external heating. High temperatures are generated locally at impact points during milling, making the process more energy‐efficient than traditional solution‐phase chemistry. The absence of solvent also enables unique reaction selectivity, faster reaction rates, and access to products not achievable in solution, while accommodating monomers with limited solubility or that precipitate under conventional conditions. Although mechanochemistry has proven more efficient than solution‐based methods for many classic reactions, its application to conjugated polymer synthesis remains underexplored, particularly in bioelectronics [104]. For instance, Zubair et al. synthesized poly(3,4‐propylenedioxythiophene) (PProDOT) mechanochemically, achieving comparable molecular weights (M_n_ of 16.9 kg mol^−1 ^and *Ð*  = 1.18 for PProDOT‐OC6 versus M_n_ of 12.4 kg mol^−1 ^and *Ð*  = 1.8 via solvent‐based oxidative polymerization) and polymer properties using only solid reagents and inert additives [105]. However, challenges remain, including controlling polymer molecular weight, which can be complicated by concurrent chain degradation under high‐impact or prolonged milling conditions, ultimately leading to shortened chains and broader dispersity [103, 104, 106].

Flow chemistry offers significant advantages for polymer synthesis by enhancing heat and mass transfer through a high surface‐to‐volume ratio. As a closed system, it enables safer operation under elevated pressures and temperatures (up to 400 °C and 200 bar), provides precise control over reaction parameters, and reduces risks associated with handling hazardous reagents and high‐energy intermediates at scale. For conjugated polymers, these features enable rapid polymerization and access to reaction conditions, such as temperature, pressure, and concentration, that are often inaccessible in traditional batch reactors. Consequently, flow chemistry opens novel process windows that can improve both efficiency and material properties [86, 97, 107, 108, 109].

Continuous‐flow synthetic methodologies have been successfully implemented in Pd‐catalyzed cross‐coupling polymerizations, including Stille and DArP, providing efficient access to well‐defined conjugated polymers with controlled molecular weights and narrow polydispersity. For example, Kim et al. [110] reported the synthesis of six donor–acceptor (D–A) conjugated polymers encompassing p‐type, n‐type, and ambipolar semiconducting materials using a customized continuous‐flow reactor. The approach enabled the preparation of polymers with high degrees of polymerization (ranging from 31.0 to 87.6) within short reaction times (≤15 min), while also demonstrating excellent reproducibility, as evidenced by a standard deviation of the degree of polymerization below 5%.

The high surface‐to‐volume ratio of flow reactors makes them ideally suited for integrating heterogeneous catalysis and photocatalysis. Immobilized catalysts and photosensitizers can be fixed within the reactor, allowing reagents to continuously flow over them. This integration offers three main advantages: it eliminates the need for catalyst separation from the product stream, greatly simplifying purification; it enables regeneration or replacement of the immobilized catalysts or photosensitizers after a number of cycles, reducing costly and wasteful disposal; and it provides enhanced mixing and improved light penetration in microreactors to increase photocatalytic efficiency and enable transformations that are unfeasible or inefficient in batch processes.

Jin et al. reported the synthesis of poly[[4,8‐bis[(2‐ethylhexyl)oxy]benzo[1,2‐b:4,5‐b']dithiophene‐2,6‐diyl][3‐fluoro‐2‐[(2‐ethylhexyl)carbonyl]thieno[3,4‐b]thiophenediyl]] (PTB7) via Stille polymerization under continuous‐flow conditions, affording the polymer with a moderate number‐average molecular weight (M_n_) of 13.9 kDa and a dispersity (Đ) of 2.8 [111]. The Researchers achieved high reproducibility, as evidenced by low experimental errors of 2.15% for Mn and 3.19% for Đ, following suppression of sulfur‐induced catalyst poisoning through the addition of triphenylphosphine (TPP) as a catalyst‐protecting agent. Furthermore, no additional purification steps were required due to the use of a flow reactor packed with 10% Pd/C, highlighting the significant potential of integrating heterogeneous catalysis with continuous‐flow systems for the synthesis of conjugated polymers. Nevertheless, catalyst reusability was not confirmed, and challenges associated with catalyst deactivation arising from Pd leaching and poisoning by tin‐containing species remain unresolved. To the best of our knowledge, no studies to date have reported the synthesis of conjugated polymers via continuous‐flow methodologies employing a fully recyclable catalytic system.

A promising route would be to move from homogeneous to heterogeneous catalysis systems. Zou et al. employed a bidentate phosphino‐modified magnetic nanoparticles‐anchored palladium complex (PdCl_2_‐Fe_3_O_4_@SiO_2_‐2P) as heterogeneous catalyst in Heck, Stille, and Suzuki polymerizations. In contrast to homogeneous palladium catalysts, such as Pd(PPh_3_)_4_, PdCl_2_‐Fe_3_O_4_@SiO_2_‐2P showed remarkable air stability, with no loss in catalytic activity for up to four weeks at room temperature, enabling its recycling seven times without apparent loss of catalytic activity. This also enabled a reduction in the amount of palladium impurity to 13 ppm with respect to over thousands ppm for homogeneous catalysts [112]. In another approach, Ye et al. replaced Pd entirely with an earth‐abundant heterogeneous copper catalyst (Cu(phen)(PPh_3_)Br) for multi‑batch DArP, enabling its reuse for up to three times while maintaining high molecular weights (M_n_ up to 24.5 kDa) and structural fidelity [113]. Although these approaches have been implemented in batch mode rather than continuous flow, they suggest that analogous catalyst‑recycling strategies could be integrated into continuous‑flow reactors, thereby paving the way toward truly sustainable, industrially scalable flow synthesis of conjugated polymers.

Moreover, continuous flow processes can be scaled predictably, e.g., by extending run times or numbering‐up reactors, without the need for extensive re‐optimization typically needed for batch scale‐up. Pfizer recently introduced an automated flow platform for the manufacturing of pharmaceuticals that represents a significant advance in reaction screening, enabling over 1,500 reactions per 24 hours with high‐resolution LC‐MS analysis and outperforming well plate‐based approaches. It offers key advantages such as avoiding solvent evaporation, improved mixing, and uniform heating, while enabling direct scale‐up from nanomole screening to micromole synthesis and ultimately to 50–200 mg scale with good‐to‐excellent yields, though it currently faces limitations with heterogeneous or biphasic systems due to inefficient mixing [114]. Collectively, these approaches represent a significant shift and inspiration toward greener, more sustainable synthesis of conjugated polymers for next‐generation bioelectronic applications.

### 2.4 Renewable Chemistry: Designing Conjugated Polymers from Greener and Bio‐Based Monomers

Sustainable monomer design is pivotal to advance circular conjugated polymers in bioelectronics, supporting the transition toward greener and more responsible materials for next‐generation devices. Many monomers currently used in organic bioelectronics are produced through multi‐step, resource‐ and energy‐intensive synthesis that relies on petrochemical feedstocks [115]. As a result, recent efforts have focused on bio‐based monomers derived from renewable sources and their integration into π‐conjugated polymer backbones [34, 115, 116]. Bio‐derived building blocks are often regarded as promising alternatives because they can reduce dependence on fossil‐based materials, lower greenhouse gas emissions, and improve biocompatibility. However, their overall sustainability is highly context‐dependent: large‐scale biomass production may require additional land, with potential impact on biodiversity and ecosystems. More sustainable production routes can be achieved by sourcing biomass from by‐products or waste streams of existing industries, such as agricultural residues, thereby avoiding competition with food supply chains and minimizing additional land‐use impacts [117].

**FIGURE 4:**
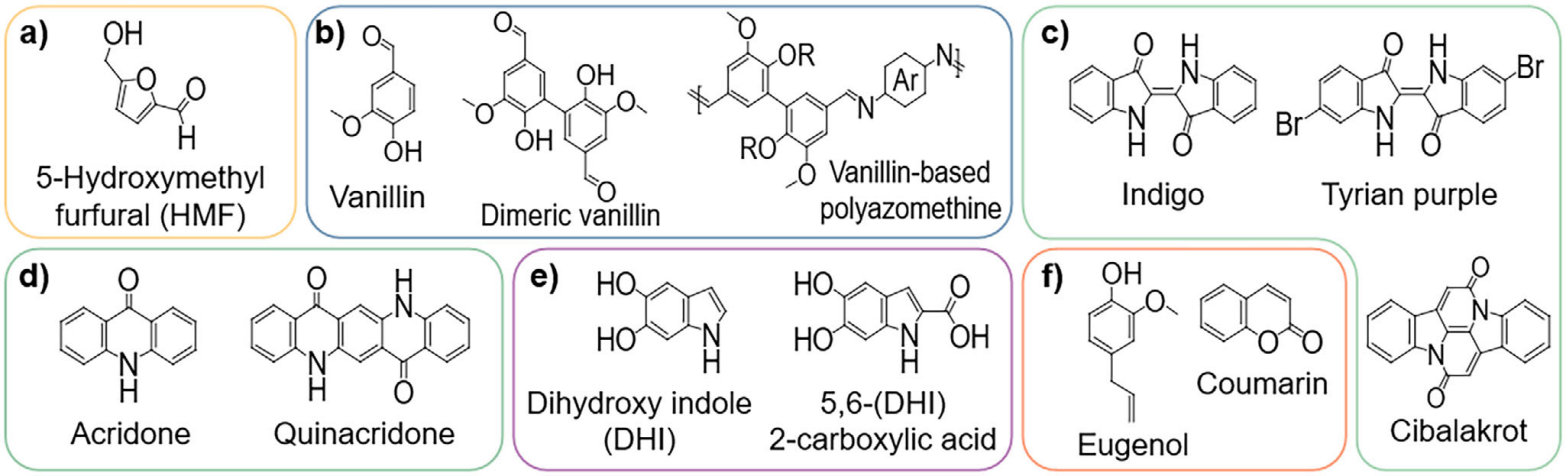
Representative classes of bio‐based and bio‐inspired monomers investigated for conjugated polymers synthesis and organic electronic applications: (a) furan derivatives obtained from biomass, including 5‐hydroxymethylfurfural (HMF); (b) vanillin and divanillin‐based conjugated motifs; (c) indigo‐derived semiconducting building blocks, including Tyrian purple and cibalackrot; (d) quinacridone‐based semiconducting structures containing the acridone core; (e) melanin‐related building blocks, including dihydroxy indole (DHI) and 5,6‐dihydroxyindole‐2‐carboxylic acid; and (f) other emerging renewable monomer platforms, such as eugenol and coumarin derivatives.

Among the most promising bio‐based monomers are furan derivatives obtained from biomass [118]. These include furfural and 5‐hydroxymethylfurfural (HMF, Figure 4a), which can be transformed into furan‐containing monomers suitable for conjugated polymers. Furan‐based units are attractive alternatives, as they can be derived from abundant renewable feedstock such as agricultural by‐products; however, they are known for their tendency to undergo ring‐opening side reactions, as they are sensitive to oxygen and light [118, 119, 120, 121]. This issue can be mitigated by stabilizing furan units through electron‐withdrawing substituents (e.g., nitro, nitrile, or carbonyl groups) or by copolymerizing with electron‐deficient units like diketopyrrolopyrrole (DPP) or benzothiadiazole (BT) [122]. For instance, Du et al. reported hole mobilities in OFETs up to 0.45 and 0.24 cm^2^ V^−1^ s^−1^ for the furan‐containing P(FDPP‐FVF) and P(ThDPP‐FVF), respectively [118].

In addition to furans, naturally derived aromatic compounds like vanillin (Figure 4b), which can be sourced from lignin in forest biomass, have gained significant attention [115, 123]. Vanillin's chemical versatility enables the synthesis of various conjugated motifs, including divanillin‐based polyazomethines building blocks (Figure 4b) [124]. These vanillin‐based polymers can degrade under alkaline conditions and in some cases produce known non‐toxic by‐products, offering environmental and end‐of‐life advantages [115, 116, 125]. However, vanillin exhibits low chemical reactivity, requiring functionalization in order to polymerize it, and electronic mobility of vanillin‐derived units is not yet competitive with conventional monomers; for instance, Dupont et al. reported a copolymer based on DPP and vanillin‐derived units that only achieved mobilities up to 6,14 × 10^−3^ cm^2^ V^−1^s^−1^ [116, 123, 124, 126].

Natural and nature‐inspired materials for organic electronic have emerged in 2010 with the seminal work form Irimia‐Vladu et al., who tested β‐carotene, indigo, indanthrene yellow G, and indanthrene brilliant orange RF as the core channel components in OFETs, obtaining field effect mobility values of 4 × 10^−4^ cm^2^ V^−1^ s^−1^, 1.5 × 10^−4^ cm^2^ V^−1^ s^−1^, ∼0.015 cm^2^ V^−1^ s^−1^, and ∼2 × 10^−3^ cm^2^ V^−1^ s^−1^, respectively [29, 34]. In 2026, Scaccabarozzi et al. showed that, by tuning the solvent and annealing conditions, β‐carotene films can exhibit mobility up to 10^−2^ cm^2^ V^−1^ s^−1^ (for β‐carotene processed from anisole and mildly annealed up to 60°C) [127].

Indigo (Figure 4c), an organic pigment that can be either synthesized or extracted from plants (primarily *Indigofera tinctoria*) through fermentation, has been widely used in organic electronics due to its intrinsic optoelectronic properties [128, 129]. Pitayatanakul et al. demonstrated an oligomer derived from the indigo derivative Tyrian purple (5,5′‐diphenylindigo, Figure 4c) exhibiting remarkable ambipolar mobilities of 0.56 cm^2^ V^−^^1^ s^−^^1^ for holes and 0.95 cm^2^ V^−^^1^ s^−^^1^ for electrons [130]. Cibalackrot (Figure 4c), synthesized from indigo, showed balanced ambipolar transport, though much lower electron and hole mobilities of 9.3 × 10^−^^3^ and 5.3 × 10^−^^3^ cm^2^ V^−^^1^ s^−^^1^, respectively [131]. Quinacridone (Figure 4d), a nature‐inspired material containing the acridone core, shows balanced ambipolar behavior when contacted with silver electrodes under N_2_, reaching electron and hole mobilities of ∼10^−^^2^ cm^2^ V^−^^1^ s^−^^1^. However, devices with gold electrode contacts (also under N_2_) display only p‐type transport, with hole mobilities around 0.1 cm^2^ V^−^^1^ s^−^^1^ [132]. Similarly, melanin and its building blocks (dihydroxy indole, DHI, and 5,6‐(DHI)‐2‐carboxylic acid; Figure 4e), whether extracted or obtained through microbial fermentation, provide a biocompatible and potentially conductive polymer framework [133, 134]. In hydrated thin films, synthetic melanin reaches conductivities on the order of 10^−^^4^ S cm^−^^1^ at 100% relative humidity, with estimated ionic conductivities up to about 30 mS cm^−^^1^ in optimized non‐functionalized derivatives, highlighting its efficient mixed ionic–electronic transport despite intrinsically modest electronic mobility [135]. Like indigo‐based materials, both melanin and quinacridone face challenges related to solubility, control over polymer properties, and effective π‐conjugation, which limit their electronic performance [136]. These issues can be mitigated by introducing thermally labile tert‐butoxycarbonyl (t‐BOC) groups, which enable processing of strongly hydrogen‐bonded structures, as demonstrated for indigo [132, 137], and quinacridone [138, 139].

Other emerging classes of bio‐based monomers include eugenol and coumarins (Figure 4f). While conductivity values have not been reported yet, their aromatic rings provide the potential for backbone incorporation, while the phenolic hydroxyl, methoxy, and allylic groups could be used to enable further functionalization [140]. Choudhury et al. reported a coumarin‐inspired monomer polymerized via Suzuki coupling to yield a fluorescent conjugated polymer [141]. Moreover, these compounds combine diverse chemical functionalities and well‐known degradation pathways, including enzymatic and photochemical routes, aligning with the demand for renewable materials designed for programmed degradability in bioelectronic applications (see Section 5 for further details about materials degradation) [115, 116].

## 3 Materials Processing and Fabrication

Materials processing and device fabrication are essential steps to integrate conjugated polymers with other components and assemble them into device architectures that can be used for applications. Organic bioelectronic devices can be developed in a wide range of form factors, such as thin films, fibers, and 3D scaffolds. In this chapter, we will focus on methods used for the processing of conjugated polymers, enabling their integration into device architectures.

**FIGURE 5:**
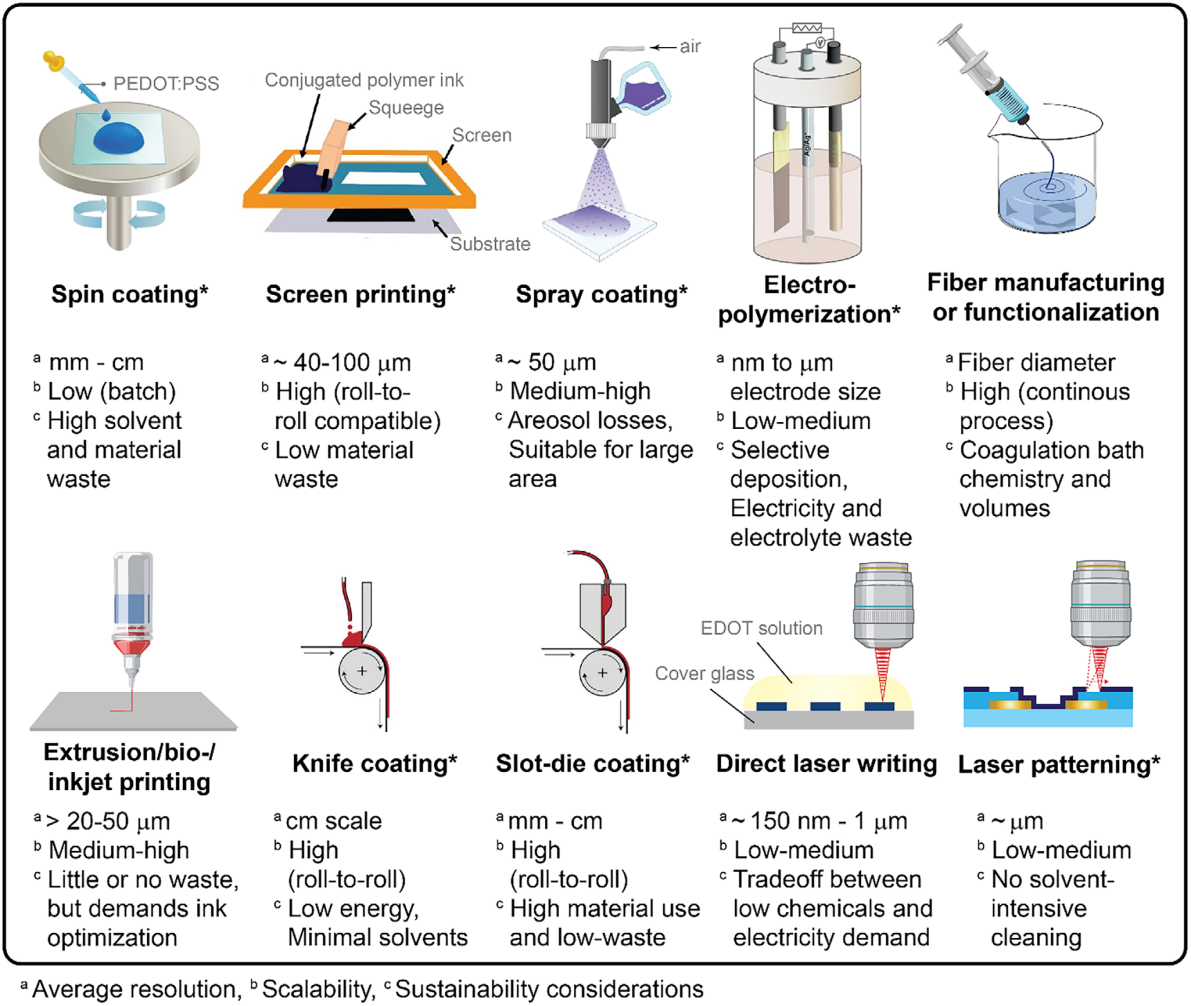
Overview of the main additive and subtractive manufacturing methods used for the fabrication of organic bioelectronic devices. The methods are reported together with their average resolution, scalability (ranked from low to high), and main sustainability considerations. ^*^Reproduced with permission [142, 143, 144, 145, 146].

One of the greatest advantages of conjugated polymers with respect to their inorganic counterpart is that they can be processed from solution at room temperature. A variety of methods have been used to process conjugated polymers in the form of thin films, such as spin‐coating, drop casting, blade coating, spray coating, and printing (Figure 5).

Coating and printing methods are scalable and generally less energy intensive than vacuum‐based techniques, which require complex high‐vacuum equipment and high electricity use for film deposition [147].

Life‐cycle and process analyses further indicate that roll‐to‐roll printing lines can reduce energy consumption per unit area by roughly 20–30% compared to equivalent sheet‐based processes [148]. Some solution‐based coating also uses significantly less active material with respect to spin‐coating. For example, Vohra et al. showed that, to coat a 2.5 × 2.5 cm^2^ area, 60 µL, 5 µL, 20 µL and 3 µL of solution are employed for spin‐coating, blade‐coating, spray coating, and push‐coating, respectively. While these values are extracted for solar cell active layer manufacturing, similar considerations could be applied for organic bioelectronic devices.

**FIGURE 6:**
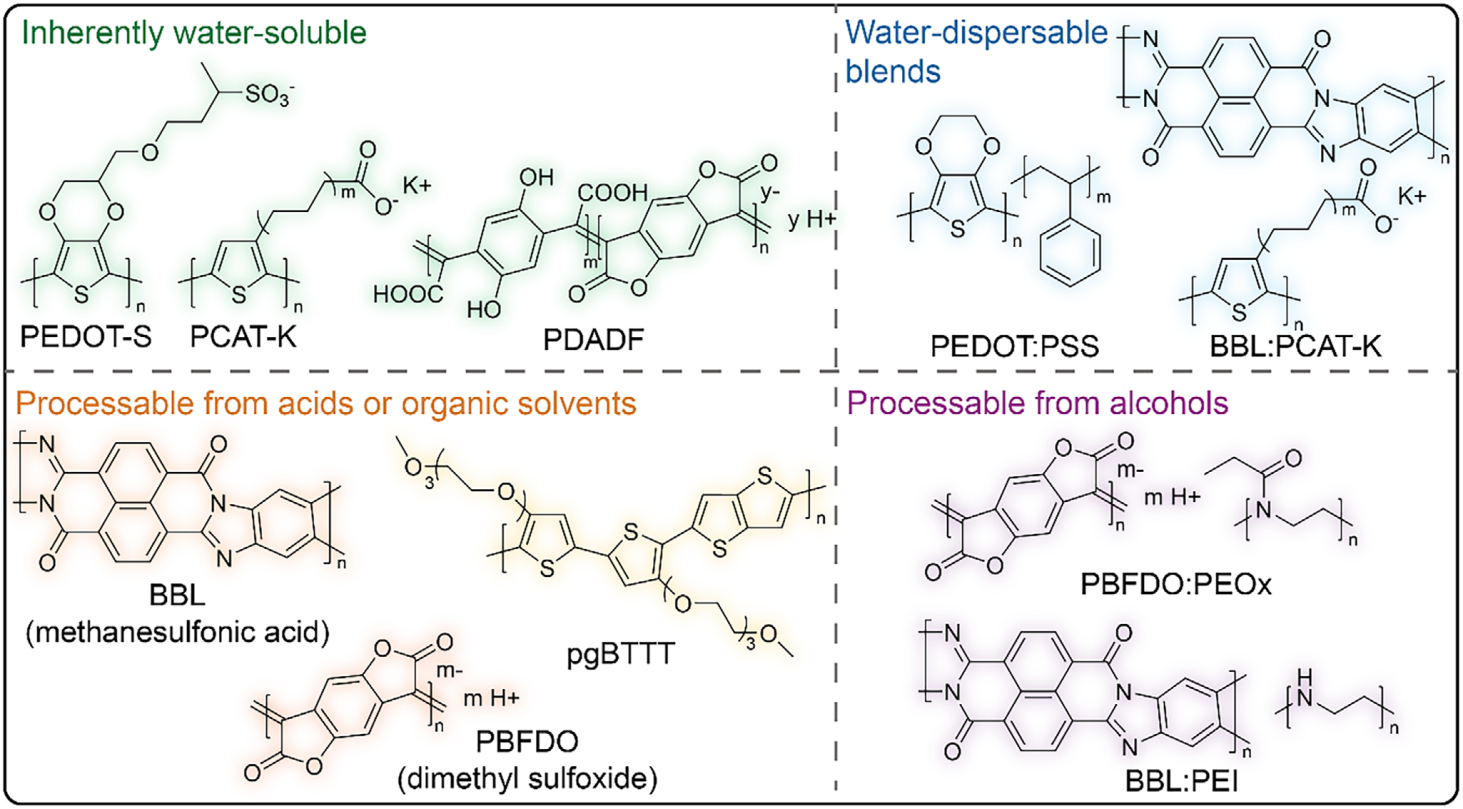
Overview of the main classes of sustainable conjugated polymer inks developed for organic bioelectronics. Conjugated polymers and conjugated polymer blends enabling processing from water, acids or protic organic solvents (such as Cyrene and DMSO), or alcohols.

While many conjugated polymers are processed from chlorinated solvents, such as chloroform or o‐dichlorobenzene (Figure 2a), some conjugated polymers can be found in the form of water‐soluble or water‐dispersible formulations (Figure 6). The concept of water‐soluble conjugated polyelectrolytes emerged in 1987 by Rughooputh et al. (Figure 1b) [23]. Water solubility is enabled by side chains with ionic terminal groups, such as sulfonate, phosphonate, and ammonium (famous examples are poly(4‐(2,3‐dihydrothieno‐(1,4)dioxin‐2‐yl‐methoxy)‐1‐butanesulfonic acid) (PEDOT‐S) [149], poly(6‐(thiophen‐3‐yl)hexane‐1‐sulfonate) (PTHS) [150], and PDADF) [151]. In some cases, the synthesis and processing of such conjugated polyelectrolytes can be performed in water. One example is PEDOT‐S, for which both synthesis and processing can be carried out in water [149]. As discussed in Section 2, Li et al. developed a new oxidant to enable the synthesis of PDADF in water, leading to a water‐processable polymer ink [89]. However, the reaction required an alkaline environment, leading to ring‐opening of 3,7‐dihydrobenzo [1,2‐b:4,5‐b′]difuran‐2,6‐dione (HBFDO) monomers and a reduction in overall electrical conductivity to around 66 S cm^−1^ with respect to over 2000 S cm^−1^ for PBFDO synthesized from DMSO [15].

Blending conjugated polymers with polyelectrolytes has led to polymer formulations with remarkable water processability and electrical conductivities. The most important example of such formulations is PEDOT:PSS, which emerged in 1995 (Figure 1b) and is arguably the most used conducting polymer in organic bioelectronic applications [152]. Ground‐state electron transfer between donor and acceptor conjugated polymers has also been introduced as a promising route to allow the processing of water‐insoluble conjugated polymers [153]. Liu et al. tested this principle with poly(benzimidazobenzophenanthroline) (BBL). While pristine BBL is only soluble in methanesulfonic acid [154], blending with the water‐soluble conjugated polyelectrolyte poly(potassium 3‐(2‐(2‐(2‐(thiophen‐3‐yloxy)ethoxy)ethoxy)ethoxy)propanoate)‐2,5‐thiophene‐diyl) (PCAT‐K) resulted in water‐soluble BBL:PCAT‐K inks, exhibiting stability for over 6 months under ambient conditions and a 10,000‐fold increase in electrical conductivity compared to pristine polymers.

A key challenge with water‐soluble conjugated polymers for organic bioelectronics is that films made from pure water‐soluble polymers tend to dissolve or delaminate when in contact with aqueous media, such as analyte solutions for chemical sensors and biosensors or body fluids for electrophysiological recordings. Therefore, these applications require strategies to form mechanically stable films that maintain integrity in contact with water‐based electrolytes. Additives and crosslinking agents are commonly added to prevent dissolution/delamination, while maintaining the properties needed for device function. Sometimes, these steps are followed by post‐processing steps, such as annealing at high temperatures to enable crosslinking (e.g., 140 °C for crosslinking with (3‐glycidyloxypropyl)trimethoxysilane (GOPS)), treatment with solvents (e.g., dimethyl sulfoxide) or acids to improve properties, such as conductivity [155]. While these processes are crucial to achieve the desired device performance, their contribution to the overall carbon footprint of device manufacturing remains largely unexplored, as most sustainability assessments of organic electronics still focus on materials selection and device architecture rather than detailed processing conditions. An example of how to solve such a challenge was presented by Schmatz et al., who introduced UV‐cleavable side chains to combine processing in water without sacrificing the performance of organic field effect transistors (OFETs) [156]. However, a major limitation for the application of this approach in OFETs was high OFF currents, leading to ON/OFF ratios on the order of 10^1^, attributed to the presence of residual water and ions within the film.

Orthogonal solvents to water enable processing from solution and film stability in aqueous electrolytes without the need for crosslinkers or post‐processing steps. However, many conjugated polymers and small molecules are soluble in chlorinated organic solvents, such as chloroform or dichlorobenzene, which are volatile and/or toxic (as discussed in section 2.2). Recently, fluorinated alcohols have also been introduced as alternative solvents for n‐type conjugated molecules [157]. The aggregation‐induced structural ordering provided by 1,1,1,3,3,3‐hexafluoroisopropanol (HFIP) has proven beneficial to the OECT mobility and volumetric capacitance of gNR films, reaching µC* values of 5.12 ± 0.13 F V^−1^ cm^−1^ s^−1^; three times higher than the value found for chloroform‐processed films (2.48 ± 0.11 F V^−1^ cm^−1^ s^−1^). Moreover, the films exhibited better electrochemical stability upon prolonged ON/OFF switching, with a 5% increase in drain current after 60 min of operation. While this is an excellent improvement in device performance, a major drawback is that these solvents are classified as PFAS (per‐ and polyfluoroalkyl substances) – a group of chemicals known for their stability and resistance to breakdown that are being phased out by many countries around the world. It is thereby unlikely that these processing conditions will ever be used for real‐world applications [158].

As new solvents are being developed, the field of bioelectronics is slowly shifting towards bio‐based, non‐toxic, and degradable alternatives (Figure 2). Deep eutectic solvents (DES) are emerging for processing conducting polymers due to their non‐toxicity, degradability, low volatility, and strong solvating ability. Their extensive hydrogen bonding networks enhance polymer solubility and enable stable dispersions, facilitating solution processing into thin films or inks for bioelectronic applications. Aguzin et al. developed PEDOT:PSS‐based eutectogels using different DES combinations, revealing tuneable ionic and electronic conductivities (up to 368 S cm^−1^) and suitability for additive manufacturing such as 3D printing of tattoo‐like electrodes for electrophysiology [159]. Makhinia et al. used dihydrolevoglucosenone (Cyrene) to process the thiophene‐based conjugated polymer poly(2‐(4,4′‐bis(2‐methoxyethoxy)‐5′‐methyl‐[2,2′‐bithiophene]‐5‐yl)‐5‐methylthieno[3,2‐b]thiophene) (pgBTTT) via inkjet printing [160]. The main challenge with this solvent is its high boiling point (around 227 °C), requiring a thermal annealing step (120 °C, time not mentioned) to dry the printed films.

Alcohols offer lower boiling points (78.5°C, 64.7 °C, 97.4°C, and 117.3°C for ethanol, methanol, 1‐propanol, and 1‐butanol, respectively) and can be produced from renewable resources like biomass or carbon dioxide (CO_2_), rather than fossil fuels [161]. Two methods have been used to develop alcohol‐based formulations: side chain engineering and blending.

Ionic/polar terminal side chains containing ammonium, hydroxyl groups, or amide groups can provide conjugated polymers with alcohol processability. However, it´s important to note that such terminal groups can be detrimental to device performance or complicate monomer synthesis (as described in Section 2) [162, 163]. For example, Ye et al. reported poly(3‐alkylamidethiophenes) (P3AAT) synthesized via DArP [162]. The tertiary amide poly(N‐hexyl‐N‐methylthiophene‐3‐carboxamide‐2,5‐diyl) (P1) showed space‐charge‐limited current (SCLC) maximum hole mobility of 2.74 × 10^−5^ cm^2^ V^−1^ s^−1^ when processed from 1‐butanol, comparable to that of the analogous poly(3‐hexylesterthiophene) (P3HET) prepared via Stille polymerization and processed from dichlorobenzene (2.09 × 10^−5^ cm^2^ V^−1^ s^−1^) [164]. No working devices could be obtained from ethanol. However, two additional synthetic steps were required for monomer amide functionalization with respect to P3HET.

Blending with insulating polymers has been used to process conjugated polymers from alcohols. Examples of such formulations are the ethanol‐based n‐type conducting ink poly(benzimidazobenzophenanthroline):poly(ethyleneimine) (BBL:PEI) [143], providing stability for at least 2 days in air and electrical conductivity up to 8 S cm^−1^.

Tang et al. developed an ethanol‐based n‐type ink based on poly(benzodifurandione) (PBFDO) and poly(2‐ethyl‐2‐oxazoline) (PEOx). PEOx stabilizes negatively charged PBFDO chains via electrostatic interactions, leading to a stable formulation at room temperature for a period of about 6 months and electrical conductivity exceeding 1000 S cm^−1^ [165]. While lower than the original 2000 S cm^−^^1^ reported for PBFDO processed from DMSO, this conductivity remains within the same order of magnitude and is likely sufficient for many applications.

Water and alcohols have a high surface tension, which can result in poor film formation. To solve this challenge, Cheon et al. introduced butyraldehyde as an additive to decrease surface tension and improve film formation of alcohol‐based dispersions of a diketopyrrolopyrrole‐selenophene‐vinylene‐selenophene (P‐DPP‐TT(7)‐SVS(3)) polymer [166]. The films showed maximum OFET mobility values of 1.03 and 0.5 cm^2^ V^−1^ s^−1^ when processed from n‐butanol and ethanol, respectively. While mobility values for chloroform were not reported, structural analysis showed that alcohol‐processed films have less edge‐on orientation of the crystalline domains compared to chloroform‐processed films.

An alternative to polymer deposition using solvent processing is electropolymerization: a method where an electric field is used to reduce or oxidize monomers from an electrolyte solution and polymerize them as a thin film onto a conductive substrate. This method enables to selectively deposit the material where needed, reducing excess polymer loss compared to spin‐coating or casting [167]. The sustainability of this process is determined by the nature of the monomers, the amount of power that is applied, and the type of electrolyte used. The main advantage is that it enables direct chemical synthesis and film formation using electricity, rather than hazardous chemical oxidants or reductants. Additionally, no purification and processing steps are needed, cutting down on the required amounts of solvents. Beyond thin films, the method has also been extensively used to develop interpenetrating conducting polymer networks for bioelectronic applications, such as electrophysiology [168]. However, it is important to note that the process is not suitable for D‐A copolymers, and requires an additional step in order to fabricate the electrode(s) onto which the conjugated polymer is deposited. The nature of the electrode material (e.g., gold, indium tin oxide, or screen‐printed carbon) can have a large impact on the carbon footprint of the whole process. However, a rigorous comparison versus solution or vapor deposition routes is still an open gap. Moreover, depending on the materials used, electropolymerization can lead to poor control over film and polymer structure, limiting batch‐to‐batch reproducibility and overall device performance with respect to other methods [169].

Additive manufacturing and printing techniques enable room temperature and high‐throughput patterning of polymers in the form of films, hydrogels, or 3D structures [170, 171, 172]. These techniques have been applied to the manufacturing of conducting polymers for various bioelectronic applications, such as electronic skin, bioinks for tissue engineering, OECTs, and electrodes for electrophysiology and sensing [171, 173, 174]. While these processes provide a route for room temperature fabrication of polymers into different form factors, they are limited by parameters such as the viscosity of the ink, nozzle size, and surface tension effects, typically resulting in feature sizes on the order of tens of micrometres. Recent process‐based LCAs of an OECT further show that fabricating metal contacts by photolithography is a major contributor to the device's environmental footprint [27]. The global warming potential (GWP100) was 5.843 kg CO_2_‐eq for lithographic Au lines versus 0.191 and 0.098 kg CO_2_‐eq for screen‐ and inkjet‐printed Ag, respectively, due to the high energy demand of cleanroom processing (vacuum thermal evaporation), the high metal consumption, and solvent‑intensive cleaning steps. Moreover, the lithographic processing steps overall contribute far more than the printed organic layers.

High fabrication resolution is important to enhance signal transduction and integration density across bioelectronic applications, such as sensing, stimulation, tissue interfacing, and neuromorphic computing—the latter demonstrated in 2015 by Gkoupidenis et al. (Figure 1a) [18]. For these applications, polymer patterning via photolithography offers sub‐micrometer resolution that cannot be achieved via conventional printing strategies. Subtractive manufacturing, such as direct laser writing using a femtosecond laser, can enable increased patterning resolution needed for micrometer‐scale patterning of conjugated polymers [146, 175]. Luo et al. demonstrated that, in addition to polymer film ablation, femtosecond laser writing can be used to directly initiate the polymerization of pure EDOT monomers, leading to 140 nm PEDOT wires with conductivity in the order of 1000 S cm^−1^ [176]. Moreover, laser micro‐annealing has been used to increase the conductivity of PEDOT:PSS thin films from 1 S cm^−1^ to around 360 S cm^−1^ without the need for additional chemicals [177]. Overall, such approaches showcase how laser writing could be used to cut down on the number of steps and solvents needed for purification and processing of conjugated polymer films.

While detailed LCAs of laser‐based patterning and annealing of conjugated polymers are still lacking, a trade‐off could exist between reduced use of chemicals and solvents and increased electricity demand, with the net environmental benefit dependent on the local electricity source.

In addition to solutions, conjugated polymers can be synthesized and processed in the form of water‐ or alcohol‐dispersible nanoparticles [144, 146]. Such nanoparticles can be synthesized via emulsification in the presence of surfactants, or via precipitation, leading to waterborne colloidal dispersions. These colloids can thereby be used to cut down toxic solvents for conjugated polymers processing and can be integrated as conductive fillers with other materials, such as biopolymers, to form composite conducting materials [178]. A critical issue that can impact both sustainability and performance for applications is the use of excess surfactants needed to prevent aggregation. Moreover, the presence of surfactants, residual monomers, and initiators requires extensive purification steps. Gwiazada et al. showed that side chain engineering with negatively charged sulfate groups, mimicking the structure of one of sodium dodecyl sulfate (SDS), can be used to stabilize the formation of nanoparticles without the aid of surfactants [179], thereby facilitating the synthesis. However, the number‐average molar mass (Mn) decreased with incorporation of the sulfate‐containing monomer, indicating a clear trade‐off of this approach.

Processing conjugated polymers in the form of fibers enables to hierarchically assemble building blocks across different scales, leading to wide design possibilities. Sustainability considerations for fiber bioelectronics have been recently discussed by Pan et al. and Shi et al. [180, 181]. Materials selection is of utmost importance as many e‐textile components have not been designed for degradability or recyclability.

Highly integrated designs provide compactness and function, but are harder to disassemble, complicating repair and recycling. Certain template materials, such as Nafion, are persistent, hard to degrade or recycle, and can produce toxic by‐products during manufacture or disposal [180, 181]. The manufacturing process can lead to large materials waste. For example, sulfuric acid coagulation baths have been used to develop wet‐spun conductive PEDOT:PSS fibers [182, 183]. Such a waste, however, could be minimized if devices were designed for long‐term or repeated use instead of single use [184]. In addition, the use of toxic solvents, energy‐intensive processing steps, and fossil‐derived polymers further increases the environmental footprint of current fiber‐based systems. LCAs are rarely applied to wearable and fiber‐based organic electronics, but recent work by Wentz et al. on integrating environmental assessment into early‐stage wearable electronics research highlights that such tools are essential to quantify impacts across raw material sourcing, fabrication, use, and end‐of‐life, and to replace unsubstantiated “green” claims with evidence‐based design choices. In line with this perspective, more sustainable pathways for fiber bioelectronics may include adopting bio‐based polymers, designing modular and disassemble architectures, minimizing the use of persistent or toxic chemicals, and considering multiple end‐of‐life options to unravel environmentally preferable alternatives (key sustainability strategies are highlighted in Table 3) [185].

**TABLE 3:** Bioelectronic applications with potential for sustainable impact together with their required operational lifetime, current end‐of‐life scenario, priority sustainability strategy, and key sustainability metrics.

Application	Required lifetime	Current end‐of‐life scenario	Priority sustainability strategy	Key sustainability metric	Reference
Point of care medicine	Single use to days	Municipal solid waste; Infectious waste; Landfill	Avoid precious metals or unsustainable sources of raw materials; Additive manufacturing, degradable or compostable materials; PFAS‐free membranes; Enable safe disposal.	GWP per test; SC index of active layer; Waste mass per unit or biodegradation/ compostability	[186, 187]
Wearables/e‐textiles	Months to years (multiple reuse cycles)	Municipal solid waste; Landfill	Design for disassembly; Washable and durable active layers; Recyclable substrates; Avoid permanent crosslinkers; Enhance biomarkers stability.	Wash‐cycle stability; Number of reuse cycles; Circularity index	[47, 181, 185]
Bioresorbable implantable electronics	Days to months	Bioresorption and clearance by the body	Additive/ subtractive manufacturing; Controlled degradation; Safe products that can be cleared by the body; Minimize non‐degradable contacts	Degradation product toxicity (LD_50_); Residual mass fraction at target timepoint; Bioresorption kinetics	[188, 189]
Sensors for food packaging	Days to months	Single‐use plastic waste; Municipal solid waste	Biodegradable or compostable substrates; Earth‐abundant contacts	Biodegradation or composting timescale; Ecotoxicity of degradation products; Material cost per unit	[190]
In vitro recordings	Variable; reuse possible with careful handling (typically 5–30 cycles depending on coating integrity)	Incineration (biomedical waste); Municipal solid waste	Replace precious metal contacts and photolithography with additive manufactured abundant alternatives; Extend reuse lifetime through surface passivation strategies	Fabrication GWP per cm^2^; Number of reuse cycles before performance loss; Solvent consumption per device batch	[191]
Environmental sensors	Days to months	Municipal solid waste; Uncontrolled release in the environment	Biodegradable or compostable substrates; Earth‐abundant contacts, materials matched to lifetime in the field	Biodegradation or composting timescale; Ecotoxicity of degradation products	[192]

Conducting polymer hydrogels [153] and 3D scaffolds are explored for bioelectronics, soft robotics, sensors, and neural interfaces due to their combination of electrical conductivity, softness, hydration, and biocompatibility. With respect to other form factors, such materials are specifically designed to be synthesized and processed from water. Moreover, their tissue‐like mechanical properties and biocompatibility make them suitable for long‐term implants or wearable devices (Section 4.2). However, they often suffer from low conductivity (up to hundreds of S cm^−1^) with respect to other form factors, high hydration, and biomolecule adsorption, making them unsuitable for applications such as high‐resolution bioelectronics, prolonged dry contact, and precision signal processing [193]. Emerging studies on conductive hydrogels highlight the need to move beyond partially degradable systems toward materials that are recyclable, fully biodegradable, or bioresorbable (Table 3). This shift is driven by an increased emphasis on dynamic cross‐linking interactions (e.g., hydrogen bonding and disulfide bonds) rather than permanent covalent networks, together with the incorporation of molecularly engineered degradable conjugated polymers [116]. We also note that incorporation of conducting polymers, depending on the synthesis route, can still drive the overall footprint, so integrating life‐cycle assessment and durability testing (e.g., reusability, self‐healing) into hydrogel device design is essential to balance performance with environmental impact.

## 4 Bioelectronic Applications

Sustainable organic bioelectronics requires materials and processes that balance environmental compatibility with key performance metrics, including conductivity, flexibility, and device stability. This chapter examines bioelectronic applications with potential for sustainable impact, discusses energy harvesters and batteries for implanted or field‐deployed devices, and highlights the limitations of academic approaches as well as often‐overlooked considerations of sustainability and commercial viability.

### 4.1 Point of Care Medicine

Point‐of‐care testing (POCT) is defined as “clinical laboratory testing conducted close to the site of patient care” [194]. Unlike traditional methods requiring sample collection, storage, and transport to specialized laboratory facilities, POCT enables rapid analysis across diverse settings. Beyond clinical benefits, POCT can ease the burden on healthcare centers, responsible for 4.4% of global greenhouse gas emissions [195], by reducing reliance on costly equipment and staff. By improving diagnostic accuracy and sensitivity, cost‐effectiveness, and ease of use, POCT devices increasingly fulfil the REASSURED (Real‐time connectivity, Ease of specimen collection, Affordable, Sensitive, Specific, User‐friendly, Rapid/Robust, Equipment‐free, Deliverable) criteria established by the World Health Organization (WHO) [196], while simultaneously contributing to several United Nations Sustainable Development Goals (SDGs) [197], including SDG 1 (No Poverty), SDG 3 (Good Health and Well‐Being), SDG 9 (Industry, Innovation, and Infrastructure), and SDG 10 (Reduced Inequalities), through a broader accessibility of high‐resolution diagnostics in resource‐limited settings.

Organic bioelectronic materials are attracting interest for wearable and POCT devices due to their potential for signal amplification (e.g., in OECTs) [198, 199]. Still, many POCTs, such as large‐scale epidemic screening, cannot be replaced by wearables. Conversely, for chronic conditions, patient‐centered healthcare increasingly demands alternatives to frequent hospital visits, where wearable bioelectronics could play a key role.

While electrochemical methods are effective for monitoring certain biomarkers with miniaturized potentiostats and remotely controlled units, such as glucose in chronic diabetes management, they still face challenges related to signal interference from complex biological matrices, electrode fouling, limited long‐term stability, and difficulties in detecting analytes with low abundance [200]. Commercial electrochemical sensors using carbon or noble‐metal electrodes, with carbon favored for its ease of printing, are widely used for glucose detection in the ±0.833 mM range [201], but are far from the range required to detect RNA (10^−15^ – 10^−18^ M). Thus, no commercial electrochemical platform achieves sensitivities comparable to the gold standard PCR, which detects 500–5000 viral RNA copies (8.3 × 10^−^^1^ ^9^–10^−^^1^ ^8^ M) with 100% accuracy [202].

**FIGURE 7:**
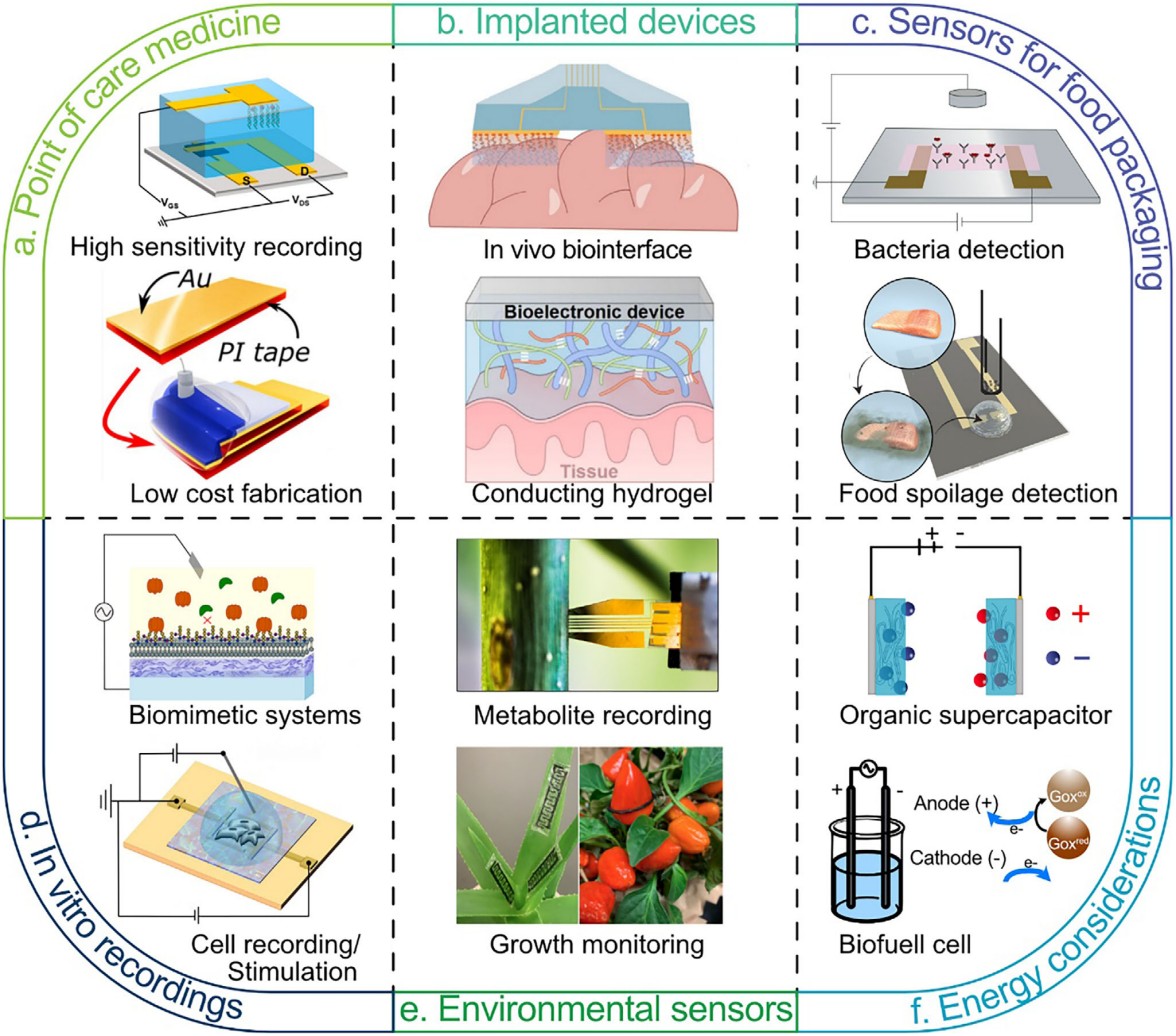
Applications where organic bioelectronics has the potential to provide a positive sustainability benefit compared with existing alternatives. (a) Point of care medicine: Ultrasensitive vertical OECT for the detection of DNA target. Reprinted from [203] with permission from Elsevier. Low‐cost fabrication approach for vertical OECTs. Adapted from [204]. (b) Implanted devices: Block brush organic bioelectronic coating with high capacitance and stability for neural interface. Adapted from [205]. Highly conductive PEDOT:PSS hydrogels with strong adhesion. Adapted with permission from [206] Copyright 2025, American Chemical Society. (c) Sensors for food packaging: OECT‐based detection of *E. coli*. Adapted from [207]. OECT for the detection of xanthine. Reprinted from [208] with permission from Elsevier. (d) In vitro recordings: Supported lipid bilayer OECT model to study the cell binding process of cholera toxins. Adapted with permission from [209] Copyright 2021 American Chemical Society. Study of human cardiomyocyte cells on an EGOFET. Reprinted from [210] with permission from Elsevier. (e) Environmental sensors: Measurements of sugar exports in Poplar trees. Adapted from [211]. Polyaniline hydrogel for plant and fruit growth monitoring. Reprinted from [212] with permission from Elsevier. (f) Energy considerations: Portable organic supercapacitor in self‐standing agarose. Adapted from [213]. Fully mediatorless organic biofuel cell combining n‐type conjugated polymer (anode) and p‐type conjugated polymer (cathode). Adapted from [214].

Electrolyte‐gated organic field‐effect transistors (EGOFETs) and OECTs provide an amplification mechanism, and several groups have pursued PCR‐level sensitivity without amplification cycles. Xu et al., for example, reported ultralow limit of detection, approaching PCR (1.07 aM) (Figure 7a) [203]. Their vertical OECT design used poly(benzimidazobenzophenanthroline) (BBL) as the active material, with ssDNA‐functionalized gates for analyte recognition, and 6‐mercapto‐1‐hexanol (MCH) as a blocking agent to prevent nonspecific adsorption.

As a step toward remote‐setting diagnostics, Ortiz‐Aguayo et al. developed a lateral flow assay integrated with an EGOFET for high‐resolution measurements [215]. The device showed the possibility to be coupled to a portable dual‐channel potentiostat while achieving a limit of detection of 10^−^^1^ ^6^ M for human immunoglobulin G (HIgG) in both clean samples and artificial saliva.

Although many organic bioelectronic sensors now achieve high sensitivity, the limited shelf life of biomarkers (approximately 7 days for aptamers and 1–3 days for antibodies) and their stringent storage requirements (e.g., −20°C) remain major limitations for home‐based applications [216]. Overall, it remains unclear whether POCT can effectively reduce the carbon footprint of healthcare systems. Ji et al. and McAlister et al. recently reported that the environmental footprint of hospital diagnostics reached 612.9 and 538 g CO_2_‐eq per test, respectively, with medical waste accounting for the largest contribution (71.3%) [217, 218]. These findings highlight the need for comparative LCA analyses between hospital‐based and home‐based testing to better guide the development of diagnostic formats suitable for remote deployment.

Furthermore, as most single‐use POCT devices are composed of plastics and complex material assemblies (e.g., membranes and electrodes), greater emphasis should be placed on the selection of degradable materials or reusable components to minimize the contribution of POCTs to medical and municipal waste streams, as highlighted in Table 3 [186, 219, 220].

### 4.2 Implanted Devices

Certain medical conditions require the sensing or stimulation of internal organs, necessitating implanted devices. Commercial systems such as pacemakers, cochlear implants, and deep brain stimulators rely exclusively on silicon or precious metals, valued for their proven stability, reliability, and biocompatibility in long‐term clinical use [221]. Yet, these rigid inorganic materials create significant mechanical and interfacial mismatches with soft biological tissues, limiting integration over time. Conjugated polymers have been explored to bridge this gap, offering mechanical flexibility, mixed ionic–electronic conduction, and soft, conformable form factors (10 MPa range versus 130 GPa for silicon) [222]. Unlike inorganic materials, however, their structures are more prone to deformation and lack the durability required for lifetime implants. Thus, the role of conjugated polymers in bioelectronic implants must differ from that of permanent implants [56, 223].

Organic bioelectronic devices and formulations offer an infinity of options for implantable systems. Yet, in seeking sustainable solutions, the key question is: what kind of sustainability do we aim for? Montesinos et al., in their scoping review, showed that for medical devices most studies focus on environmental sustainability (39.1%), followed by economic sustainability (37.9%) and social impact (23%) [224]. Moreover, the Authors point out that while most studies focused on the design, development, manufacture, and use of medical devices, more attention should be given to the end‐of‐life management and social dimension.

For temporary implants, both social and environmental sustainability aspects can be addressed by incorporating bioresorbable materials into implanted devices [225]. Implantable bioresorbable systems eliminate the need for secondary extraction surgeries, thereby reducing patient risk and healthcare burden. In addition, they can support recovery by minimizing chronic inflammation responses and enabling continuous monitoring of the healing process, allowing timely therapeutic intervention. Postoperative complications, such as infection or bleeding, can arise unexpectedly, with serious consequences; for example, approximately 20% of cardiac surgery patients require readmission due to such issues [226, 227]. Implanted sensors could monitor blood pressure in reconstructed vessels, while brain recordings may assist recovery after brain surgery or traumatic injury [228, 229]. Transient electronic devices can also provide temporary therapies, including cardiac support, localized drug delivery, or tissue regeneration [230].

From an environmental perspective, bioresorbable materials also offer a solution to a growing issue associated with medical technologies: the accumulation of electronic and biomedical waste. Conventional devices are often difficult to recycle due to their complex architecture. Moreover, devices that have come into contact with biological fluids carry a risk of biohazard contamination and are therefore typically treated as medical waste (i.e., incinerated). Bioresorbable devices can significantly contribute to reducing such waste while improving patient outcomes [231].

While bioresorbable materials such as sutures, stents, and localized drug‐delivery platforms are already in clinical use [232], short‐term electronic implants remain unavailable. Existing transient electronics often use inorganic materials (e.g., zinc, magnesium, iron) that dissolve in water, generating metal hydroxides and hydrogen gas that may pose risks to the patient, or poorly soluble oxides [233]. While other inorganic alternatives have been explored (e.g., molybdenum, tungsten and composites), organic (semi)conductors are emerging as a promising complement. Yet engineering safe and controlled bioresorption while maintaining high performance remains a significant challenge, as discussed in Section 5.

Historically, conjugated polymers were first introduced as coatings to lower impedance in biorecordings and neuronal interfaces, where improved signal transduction and mechanical matching enhanced the signal‐to‐noise ratio of the recorded signals [234, 235]. Since then, the field has evolved to introduce active device architectures, such as transistors, which enable amplification at the expense of increased power requirements and circuit complexity, as well as more advanced closed‐loop systems that integrate sensing, signal processing, and therapy [236].

These increasingly sophisticated device architectures also necessitate reliable power sources and data transmission modules, further complicating system design, integration, and, ultimately, end‐of‐life management in the context of transient or bioresorbable electronics.

One strategy to simplify implanted bioelectronics is to polymerize conjugated polymers directly within living organisms.

Several methods have been proposed to achieve in vivo conjugated polymer formation using endogenous cellular activities or external triggers. For example, Ouyang et al. demonstrated the electrodeposition of ∼500 µm PEDOT clouds in the rat hippocampus, reducing electrode impedance from over 100 kΩ to 33.6 ± 1.4 Kω [237]. Zhang et al. further showed that genetically targeted expression of horseradish peroxidase at the neuronal surface can drive the localized polymerization of polyaniline in vivo [238]. More recently, Samal et al. demonstrated that hemoglobin can trigger the polymerization of HBFDO, enabling the deposition of an n‐type conjugated polymer within living organisms without the need for external oxidizing agents [239]. Together, these studies provide compelling examples of in vivo tissue and cellular modification through conjugated polymer formation. However, the fate, toxicity, and long‐term impact of these polymerized materials remain to be established [240].

A critical challenge towards clinical translation is the systematic validation of new materials and device concepts within existing regulatory frameworks. Strict requirements, such as the EU's Medical Device Regulation (MDR 2017/745) together with associated standards (such as ISO 10993: Biological Evaluation of Medical Devices), can pose significant barriers towards innovative and potentially more sustainable medical technologies by increasing complexity [224], cost, and uncertainty around approval [241, 242]. Current regulatory frameworks focus primarily on patient safety and device performance and do not yet explicitly incentivize the use of sustainable materials or circular economy principles, thereby creating a challenging regulatory environment for circular design [243]. To date, PEDOT:PSS remains the only organic electronic material approved by the Food and Drug Administration (FDA) and bearing the CE mark for use in human clinical trials, with bioelectronic devices mainly targeting brain interfaces [152].

### 4.3 Sensors for Food Packaging

The global food supply is rapidly expanding, growing at 6.7% annually and valued at $9.12 trillion. At the same time, food safety remains a major concern, with nearly 1 in 10 people affected by foodborne illness each year, around 600 million individuals, leading to approximately 420,000 deaths [244]. Expiry dates help prevent food poisoning by indicating when food should no longer be consumed. Simultaneously, the United Nations emphasizes the need to reduce global food waste, as roughly one‐third of the food supply, about 1.3 billion tons, is lost annually [244]. In this context, sensors integrated into packaging offer a route to assess actual product quality beyond conservative date labelling, potentially improving both safety and resource efficiency, in line with SDG 12, Target 12.3 [245].

Food spoilage arises from microbial colonization or intrinsic degradation processes, such as enzymatic activity and chemical reactions. These changes alter the food environment, shifting composition and creating consumption risks. Sensors tailored to specific food types (e.g., poultry, milk) can detect compositional shifts indicative of microbial contamination. A common metric is microbial load, expressed as colony‐forming units (CFU) per milliliter, gram, or square centimeter. Health risks become critical when counts reach approximately 5–6 log CFU per unit [246]. Traditionally, detecting and quantifying microorganisms requires sending samples to specialized laboratories for plate culture or ATP luminescence assays. Disposable sensors can monitor microbial growth, but they are often organism‐specific, requiring different sensors for each food type. He et al. and Vizzini et al. demonstrated that organic bioelectronic sensors can detect bacterial contamination linked to food spoilage, reaching a detection limit of 4 log CFU, sufficient to raise strong suspicion of toxicity (Figure 7c) [207, 247]. However, these sensors must be placed in direct physical contact with food either directly or indirectly (e.g., through the use of microfluidics) [248].

One strategy for large‐scale deployment of organic bioelectronic devices is to detect food spoilage independently of its underlying cause. Following this approach, ATP degradation products such as xanthine and hypoxanthine have been proposed as robust spoilage markers in fish and meat (Figure 7c) [249]. Lin et al. recently developed an accumulation mode OECT‐based sensor that could detect xanthine and hypoxanthine across a wide concentration range, from 3.8 mg·100 g^−^^1^ on day 0 to 30 mg·100 g^−^^1^ after six days of degradation [208]. However, direct sample sensing was not yet possible, as samples needed homogenization in water, requiring laboratory equipment. Additionally, the interferent‐screening layer was made of Nafion, a PFAS‐classified material, demanding alternative materials (as further discussed in Section 6.1) [45].

In contrast to bacteria and nonvolatile chemical markers, sensors targeting volatile organic compounds (VOCs) released during spoilage (e.g., volatile amines, hydrogen sulfide) can operate in the packaging headspace and therefore do not require direct contact with food. The advantages and limitations of food spoilage detection using microbial VOCs have been comprehensively reviewed by Ramadan et al. [245].

Electronic noses (E‐noses) are a promising sub‐class of such sensors, operating as arrays of gas sensors coupled with data‐processing approaches, such as artificial neural networks, to detect and identify odor signatures from food‐related compounds [250]. Several E‐nose systems are already implemented in the food industry; for example, the EOS835 is an advanced electronic nose used for the early detection of bacterial growth in coffee beans [251].

Conjugated polymers have been shown to exhibit reversible responses in the presence of specific gases, making them promising candidates for VOC detection. In these systems, gases are not only physically absorbed but also chemically interact with the polymer, altering its doping state and, consequently, its conductivity. Such reversible sensing behavior is particularly attractive for long‐term applications, such as continuous monitoring inside transport trucks or storage facilities with frequently renewed cargo. To simplify device architectures, Kim et al. developed an E‐nose strategy based on a single conjugated polymer active material while tuning the chemical structure of the poly(vinyl alcohol)‐based membrane [11]. The resulting sensors displayed distinct responses toward VOCs (i.e., methanol/ethanol), enabling the generation of characteristic gas fingerprints. Furthermore, Anisimov et al. demonstrated a proof‐of‐concept E‐nose platform for food spoilage detection using four different OFET configurations [250]. The system was trained to recognize VOCs produced during meat decomposition under varying humidity conditions, allowing reliable gas identification across diverse environmental conditions.

From a regulatory perspective, sensors integrated in packaging must comply with stringent regulations to ensure that sensor materials themselves do not contaminate the food (e.g., regulation (EC) No 1935/2004 in the EU). In practice, this typically implies that sensor components must either be made from food‐grade materials or be encapsulated so that no substances can migrate into the food under use. It is also important to note that such regulatory frameworks vary considerably between countries and regions, meaning that a device designed for one market may require substantial redesign, revalidation, or additional certification before it can be legally commercialized elsewhere [245].

Another main challenge is the complexity of real food matrices, leading to signal drift, poor reproducibility, or false positives. To date, there are no widely accepted standardized metrics or test methods for VOC‐based sensors, highlighting the need for standards that enable comparison between products and clearly define sensor performance in terms of sensitivity, specificity, response time, and rates of false responses. Coupling materials and device innovations with AI‐based data analysis could increase data robustness by extracting meaningful patterns from complex VOC signatures, compensating for drift and matrix effects, and improving the identification accuracy of spoilage states across diverse food products [252].

Cost also remains a major limitation in the development of sensors for food spoilage monitoring, as only a limited number of food products possess sufficient value to justify the use of sensors incorporating materials with high synthetic complexity and relatively high value (such as conjugated polymers). Consequently, disposable sensors may only be viable for high‐value products rather than widespread deployment; for instance, DNA recognition molecules can cost around $500 for 0.1 mg [244].

In addition, locally powering individual devices for each food package would introduce installation and manufacturing costs that substantially exceed the 3%–15% of product value that companies are typically willing to allocate to packaging [253]. This makes battery‐powered or otherwise actively powered sensing economically challenging, further motivating passive indicators or self‐powered device architectures.

Finally, future food‐integrated sensors will also need to balance regulatory and performance requirements with end‐of‐life considerations, such as recyclability of packaging and the avoidance of persistent materials (e.g., PFAS‐based components as highlighted in Table 3).

### 4.4 In Vitro Recordings

The interface between living tissues and organic bioelectronic devices enables these materials to serve as seamless platforms to interrogate biological systems, including cells and tissues. With an early example reported in 1994, in which Wong et al. demonstrated that the oxidation state of polypyrrole influences the culture of mammalian cells (Figure 1a) [20], the field has progressively moved toward the development of in vitro models aimed at supplementing, or ultimately replacing, animal testing, as physiological differences between animal models and humans often limit the predictive value of in vivo studies. Consequently, the global market for organ‐on‐chip technologies (one of the many in vitro technologies) is projected to reach $303.6 million, corresponding to a growth of 39.9% by 2026 [254].

The limitations of organic bioelectronic materials, as well as their potential to overcome current bottlenecks, have been comprehensively reviewed by Pitsalidis et al. [255]. Broadly, in vitro approaches aim to replace, reduce, and refine animal testing while improving the reliability of drug screening through the use of human cell lines and organoids [256]. This transition also enables higher‐throughput drug discovery. In line with these advantages, substantial investments continue to be directed toward this field, particularly as drug testing phases can reach average costs of approximately $114.2 million (as an average of 10 pharmaceutical firms), including animal studies [257]. This approach also substantially improves the ethical aspects of such studies by reducing the number of animals required per experiment.

For example, preclinical cardiac safety testing still relies heavily on animal studies, especially telemetry measurements in dogs and non‐human primates, as required by regulatory guidelines (i.e., ICH S7B), despite recognized species differences in cardiac electrophysiology and QT‐rate correction that weaken translation to humans. A typical ICH S7B study requires a minimum of 4–6 animals across dose escalation, including controls [258]. However, some studies use more. For example, a recent study employed 112 dogs and 48 non‐human primates to produce a protocol for QT risk telemetry [259].

Low‐throughput techniques, such as single‐cell patch‐clamp, provide intracellular information on the action potentials of individual cells. In contrast, high‐throughput techniques (e.g., microelectrode arrays) enable simultaneous, long‐term monitoring of large populations of cells, which is particularly relevant for drug screening, cardiotoxicity and neurotoxicity assessment, and safety pharmacology studies [260].

Since the early emergence of organic bioelectronics, conjugated polymer coatings have become a widely adopted approach to enhance biological signal transduction in vitro [255]. However, such devices are commonly fabricated using precious metals patterned via photolithography, substantially increasing their environmental footprint. Garma et al. demonstrated that inkjet printing can be used to fabricate PEDOT:PSS‐based multielectrode arrays with individual electrode areas of 6.50×10^−2^ mm, corresponding approximately to circular electrodes with a diameter of 300 µm [260]. The electrodes exhibited signal‐to‐noise ratio (SNR) of 10.2 dB and an average impedance of 19.5 kΩ at 1 kHz, well below the 160 kΩ reported for commercially available gold MEAs. However, the recording capabilities of planar MEAs are generally limited to extracellular field potentials.

Organic transistors have been introduced to improve sensitivity and temporal resolution of in vitro recordings [261]. For instance, Kyndiah et al. demonstrated that EGOFETs based on P3HT enable a high signal‐to‐noise ratio (60 dB) for the non‐invasive recording of cardiomyocytes action potentials (Figure 7d). The Authors used the action potential duration (APD)‐shortening drug Nifedipine (NIFE) and the APD‐prolonging drug E‐4031 to benchmark the platform against the patch clamp gold standard and demonstrate its efficacy for pharmacological screening of drugs. A major difference was in the measurement duration, which was constrained to 10 minutes for patch clamp, but could be extended to 30 minutes with EGOFETs [210]. However, these transistor configurations still rely on gold source and drain electrodes fabricated by photolithography and vacuum deposition, processes associated with high embodied energy and significant environmental impact (as discussed in Section 3 and highlighted in Table 3). Gold in particular carries one of the highest embodied energy values among electrode materials used in organic electronics (200´000 GJ t^−1^ Au) [262], and its extraction and processing contribute disproportionately to the lifecycle carbon footprint of devices. Replacing gold contacts with solution‐processable, earth‐abundant alternatives, such as conducting polymer‐based or printed carbon electrodes, while maintaining the high conductivity (≥10^2^ S cm^−1^) [263], and micrometer resolution of photolithography‐patterned electrodes remains an open challenge, but represents a critical step toward fully sustainable organic bioelectronic platforms.

### 4.5 Environmental Sensors and Plant Organic Bioelectronics

The properties of conjugated polymers make them well‐suited for direct contact with living tissues, including plants. Low‐power, environmentally friendly devices deployed in agricultural settings can provide valuable data to optimize farming practices and advance fundamental understanding of plant biology [264]. Field data could enable farmers to reduce water usage and selectively treat only plants affected by pests.

Kim et al. fabricated vapor‐deposited organic bioelectronic electrodes to monitor plant responses to water stress and UV exposure using bioimpedance spectroscopy, probing the integrated electrical properties of both the electrode interface and the plant's internal tissues [265]. Hsu et al. developed conjugated polymer hydrogel memristors incorporating graphene oxide nanoparticles to monitor growth: the devices exhibited internal resistance changes accommodating up to 200% growth, while simultaneously storing sufficient charge (2330 mF·cm^−^^2^) to power LEDs (Figure 7e) [212]. Other approaches integrate sensors directly into plants. Diacci et al. inserted OECTs functionalized with sucrose‐degrading enzymes into poplar trees to track sap dynamics using a portable Arduino unit (Figure 7e) [211].

Despite this versatility, most of these sensors still rely on precious metals for electrical contacts, and their environmental fate is rarely considered. Yang et al. explored degradable designs by fabricating ultrathin OFET sensors on dextran films (239 nm), which dissolve harmlessly into plant nutrients after 30 min immersed in the soil or 10 seconds exposed to rain on a leaf; however, the active semiconductor dinaphtho[3‐b:2′, 3′‐f]thieno[2‐b]thiophene  (DNTT) is not degradable, raising potential long‐term toxicity concerns [266].

While conjugated polymers enable monitoring of key environmental parameters, such as plant metabolites and growth, little emphasis has been placed on translating laboratory measurements to field applications. For example, device lifetimes remain far from sufficient to match the timescales required for monitoring plant growth and pathogen emergence in precision agriculture applications [267]. To date, growth monitoring has generally been limited to measurements spanning hours to days, with Wang et al. reporting a record device elongation capability of 20 mm [268], which remains insufficient for tracking an entire plant growth cycle. Similarly, pathogen sensors based on EGOFETs exhibit limited operational lifetimes, restricting their use in long‐term wearable configurations and therefore still requiring plant samples to be collected and transferred to laboratories for preparation and analysis. In parallel, materials capable of controlling device degradation rates are needed to prevent unnecessary environmental pollution (see Table 3). Promising directions have recently been demonstrated by Atreya et al., who employed beeswax blends to tune device transience over operational lifetimes ranging from 10 to 100 days [269]. Devices operating within these timescales could significantly expand the practical potential of plant bioelectronics.

Considerations of material cost and on‐site deployment are often overlooked in environmental sensor research. For plant monitoring in particular, bulky instrumentation is impractical for field applications, highlighting the need for miniaturized sensing platforms capable of performing measurements directly on site [265]. Material cost represents an additional barrier to large‐scale adoption. For example, the high‐performance semiconductor DNTT exhibits mobilities of up to 2.1 cm^2^ V^−^^1^ s^−^^1^, [270] but costs approximately $2,500 g^−^^1^. In contrast, quinacridone‐based semiconductors cost around $70 g^−^^1^ while still providing mobilities of ∼0.01 cm^2^ V^−^^1^ s^−^^1^, which are sufficient for many sensing applications despite being two orders of magnitude lower [271]. Matching material performance to application‐specific requirements, rather than pursuing maximum performance alone, could therefore substantially reduce costs and facilitate large‐scale deployment.

### 4.6 Energy Considerations

Implanted sensors, food packaging sensors, and environmental sensors hold great promise for organic bioelectronics, but only if questions of power supply and data collection are addressed. Current implantable standards, such as pacemakers and cochlear implants, rely on lithium‐ion batteries for their high energy density and reliability [143].

Organic electronic supercapacitors store energy through both electrical double‐layer effects and pseudocapacitance, in which ions are adsorbed and desorbed from conjugated polymers (Figure 7f). Compared to batteries, supercapacitors offer higher power density but deliver current at variable potential. Recent advances enable >10 000 charge–discharge cycles with specific power up to 10^2^ W g^−^^1^ [213]. Nonetheless, they remain less stable than inorganic capacitors, which can endure >1 000 000 cycles versus ∼10 000 for current organic designs, limiting their applicability to long‐term implantable bioelectronics.

Power supply also remains a major challenge for food‐packaging sensors, as conventional batteries are often too costly and bulky for large‐scale packaging applications. As a result, RFID‐connected sensors are increasingly favored because they can operate in a passive mode and be wirelessly read using smartphones [272].

Living organisms provide chemical energy that can be harvested using enzymatic biofuel cells (Figure 4f), which typically generate ∼1 V, the most common example being glucose biofuel cells [273]. Their current output, however, is limited by local fuel availability; for example, glucose levels in blood are around 5 mM and oxygen concentrations in tissue about 50 µM [274]. In the field of environmental sensing, several studies have demonstrated the integration of biofuel cells within fruits, where glucose concentrations can reach approximately 10 mM [275, 276].

To date, all‐organic biofuel cells have only been demonstrated by Ohayon et al. Their proof‐of‐concept device, based on p(EDOT‐co‐EDOTOH) (EDOTOH = hydroxymethyl 3,4‐ethylenedioxythiophene) and an n‐type polymer based on naphthalene tetracarboxylic diimide units (NDI‐T2), retained 40% of its initial power output after 3 days and achieved a maximum power density of 2.8 µW cm^−^^2^ at 10 mM glucose [214]. Comparable power densities have also been reported for microbial fuel cells, which employ whole microorganisms as catalytic centers [277]. While such biofuel cells are unlikely to power energy‐demanding implants such as pacemakers (10–30 µW) or retinal prostheses (∼250 mW), their output is sufficient for low‐power organic bioelectronic devices, including electrochemical transistors (∼1 µW) [278], and organic electronic ion pumps for drug delivery (∼10 µW) [279].

Beyond biofuel cells, temperature gradients can also generate electricity using conjugated polymers. A thermoelectric device consists of two legs, one n‐type and one p‐type; a temperature difference between the leg extremities drives charge carriers in opposite directions, creating a voltage gradient that drives current, with the Seebeck coefficient defining the voltage per unit temperature gradient [280]. Conjugated polymers have long been of interest for thermoelectrics due to low‐cost and scalable production. However, until the recent emergence of stable n‐type semiconductors such as naphthalene diimide, most thermoelectric materials were only half‐organic. Power factors for p‐type polymers (PEDOT:PSS or DPP‐based polymers) typically range from 100–300 µW m^−^^1^ K^−^^2^ [281], while only recent thiophene‐fused benzodifurandione oligo(p‐phenylenevinylene) reached a similar power factor for n‐type materials [282].

In parallel, self‐powered technologies such as triboelectric nanogenerators (TENGs) and piezoelectric generators (PGs) have been introduced to harvest energy from motion, either through contact electrification and electrostatic induction generated by contact between two materials with different triboelectric polarities, or through the deformation‐induced polarization of a dielectric material under an applied pressure, respectively [283, 284, 285, 286]. TENGs provide much higher power density at a low‐frequency range (below 4 Hz, typical of human motion) with respect to PGs [287, 288], but they exhibit poor performance in humid environments and are prone to material degradation under repetitive contact. Both device types have been used to power organic bioelectronic devices for biomedical implants or agricultural applications. For example, piezoelectric generators have been coupled with either magnetoelectric or ultrasound transduction to enable wireless powering of stimulators for neuromodulation in vivo [289]. Moreover, TENGs have been used to harvest mechanical energy from wind‐induced leaf motion to power organic electronic ion pumps used for the delivery of agricultural chemicals to plants [290].

Both TENG and piezoelectric generators can be made using biodegradable and bioresorbable materials, enabling powering in temporary biomedical implants or agricultural sensors [291, 292]. However, both classes of devices currently suffer from limitations such as low electromechanical output and current, power management challenges, and limited structural stability and durability under physiological conditions [293]. For a more detailed comparison of performance and fit for applications, readers are referred to the following reviews [284, 285, 286, 294].

Conjugated polymers have been integrated either as composite blends within the core triboelectric or piezoelectric active layer, or as charge‐trapping/charge‐transfer layers to improve the performance of PGs and TENGs [295, 296, 297, 298]. Moreover, supramolecular assemblies of conjugated monomers have, in some cases, been shown to exhibit piezoelectric properties [295, 299]. While these examples highlight a promising opportunity to use conjugated polymers to improve the performance of self‐powered energy‐harvesting devices, the resulting multi‐component composites and blends complicate end‐of‐life management, such as separation and recycling. In this respect, biodegradable or recyclable materials could offer a possible route to ensure that performance gains do not come at the expense of long‐term sustainability.

## 5 Degradable Organic Bioelectronics

### 5.1 What Is Transient Bioelectronics?

Transient electronics have emerged as a solution for applications that are short‐lived and can degrade, physically disappear, dissolve, resorb, or disintegrate after a defined period of stable operation [300]. Since the appearance of the first transient electronics in 2012, the field has rapidly evolved but remains relatively new [301]. Such applications range from implantable medical devices [302, 303, 304, 305], to environmental sensors [306, 307, 308], and hardware‐secure devices [309, 310]. For example, transient sensors and drug delivery systems can be implanted into the body to diagnose or treat illness and degrade after use, eliminating the need for surgical removal and reducing the risk for infections and complications. Transient devices can also help to reduce waste generated by the growing use of disposable point‐of‐care diagnostics, e‐skin devices, wearables, and sensors, whose complex architectures make disassembly, recycling, or safe disposal challenging [186].

**FIGURE 8:**
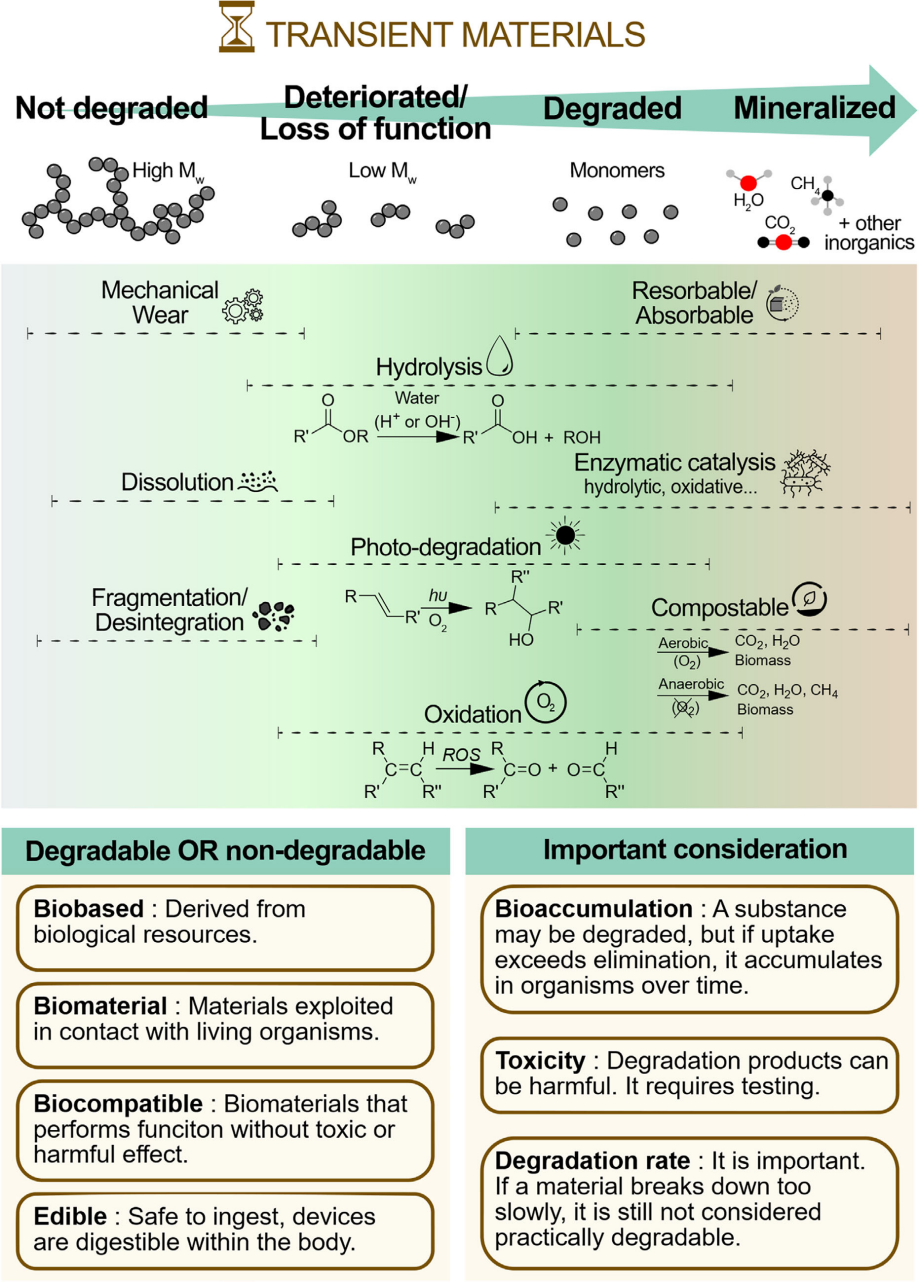
Overview of degradation mechanisms and key terminologies in transient materials. The upper panel illustrates the progression from intact polymer structures (high M_w_) through deterioration (loss of function, low M_w_) and degradation (monomers) to complete mineralization (CO_2_, H_2_O, and inorganics). The middle panel shows the main mechanisms in which a material undergoes changes in polymer structure. The lower panels define key terms distinguishing degradable from non‐degradable materials (biobased, biomaterial, biocompatible, edible) and highlight critical considerations for transient device design (bioaccumulation, toxicity, and degradation rate).

Several terms are used to describe transient electronics due to their broad definition, such as degradable, disintegrable, and resorbable, leading to inconsistent terminology. Moreover, the definitions of these terms often vary across disciplines, and many are used interchangeably or even misused, resulting in a lack of uniformity in the literature. In an attempt to unify the field, we have listed and defined the different terms in Table 4. The relationships between these terms are visualized in Figure 8.

**TABLE 4:** Definition of the different terms related to transient bioelectronics, including possible standards for assessment. Terms have been defined according to the International Union of Pure and Applied Chemistry (IUPAC) definition, the International Organization for Standardization (ISO), or from literature.

Term	Definition	Relevant standards for assessment
Transient	Devices that can degrade, physically disappear, dissolve, resorb, or disintegrate after a defined period of stable operation.	No metrics
Biobased	Materials that are composed of biological products originating from the biomass (including animals, plants, and marine or forestry materials) [311].	ISO: 16620 ASTM: D6866‐24a
Biomaterials	Materials that are used in contact with living organisms, microorganisms, or tissues [312]. It should be noted that those can be both synthetic or natural.	ISO: 10993 ASTM F756, F2027
Biocompatibility	Ability of a material to interact with living systems and perform its function without eliciting toxic or injurious effects on biological systems [312].	ISO: 10993, 18562, 22442 ASTM: F748, F749, F2475, F756, F895, F1904, E2524
Degradation	(1) Progressive loss of the characteristics or of the performance of a material or a device [312]. (2) For biorelated polymers: Degradation that results in desired changes in the properties of the materials caused by macromolecule cleavage or molar mass decrease [312].	ISO: 4892 ASTM: D5208, D3045
Biodegradation	Degradation of a material due to biological activity. A degradable polymer is not necessarily biodegradable, whereas a biodegradable polymer is always degradable. A degradation in vivo that results only from water or else without contribution of living elements cannot be considered biodegradation [312].	ISO: 17556, 14855, 22403, 22404, 23977, 846, 19679 ASTM: D5338, D5511, D5209, D5988, D6691, D5526, D5929
Aerobic biodegradation	Biodegradation in presence of molecular oxygen [312].	ISO 17556, 14855, 14852, 19679, 22403, 22404, 23977 ASTM: D5209, D5338, D5988, D7475, D6691
Anaerobic biodegradation	Biodegradation in the absence of oxygen [312].	ISO: 15985 ASTM: D5511, D5526
Compostable	Material that undergoes degradation by biological processes *during composting* and leaves no visible, distinguishable, or toxic residue [313]. *Composting* is a process where biological decomposition of organic matter occurs due to microorganisms (usually bacteria and fungi) [312]. It requires very specific environment i.e., warm temperatures, nutrients, moisture, and oxygen.	ISO: 17088, 14855 ASTM: D6400, D5338, D6868 EN: 13432, 14995
Bioassimilation	Conversion of a material into biomass (substances produced by microorganisms, plants, or animals) [314] via biochemical processes [311].	ISO/TS 4988
Bioresorbable	A compound or a device that can be totally eliminated or bioassimilated by an animal or a human body through natural pathways. Some polymers can be bioresorbed after an initial hydrolysis [311].	ISO: 37137 ASTM: F2902‐24, F3160‐21
Bioabsorbable	This term is often used interchangeably with “bioresorbable” although no definition can be found in IUPAC. In some literature, the terms have different meanings, for example, bioabsorbable refers to polymers that can be *dissolved* in biological fluids, while bioresorbable denotes polymers that can be converted under physiological conditions into degradation products that will be resorbed inside the body [315].	ISO: 37137 ASTM: F2902‑24, F2502, F1925‑09, F2313‑10, F2579‑11, F3160‐21
Mineralization	Process through which an organic substance becomes impregnated by or turned into inorganic substances [312]. In the case of polymer biodegradation, mineralization is used to reflect conversion to CO_2_, H_2_O, and other inorganics. CH_4_ can be considered part of the mineralization process because it forms in parallel with mineral products during anaerobic composting, also called methanization.	ISO: 14855, 17556, 15985, 11734 ASTM: D5338, D5988 EN: 13432
Fragmentation	Breakdown of a material into particles. The size of the fragments or the breakdown mechanism does not matter [312].	ISO: 16929
Disintegrable	Materials that can be fragmented into particles of a defined size [312]. Biodisintegration should be used when the fragmentation results from the action of cells.	ISO: 16929, 22766 ASTM: D8618, D8619 EN: 17428
Dissolvable	This can be said for a material that can undergo *dissolution*, e.g., homogeneous phase formation (solution) obtained from the mixing of two initial phases [316]. So, a material that is dissolvable is a material that can form a solution in a solvent. When referring to transient electronics, it would relate to a material, e.g., polymer or metal, that can dissolve in physiological fluids.	ISO: 20721
Hydrolysis	This can be said for a material that *hydrolyzes*. The material being hydrolysed reacts with water leading to the breakage of one or more bonds [317]. Hydrolysis involves a chemical reaction, while dissolution refers to a physical reaction. Hydrolysis is a type of degradation (hydrolytic degradation).	
Edible electronics	These are devices that are safe to ingest, fully digestible within the body, and that can be safely released into the environment after usage without the need of recollection. Such devices are either digested or metabolized (processed by the metabolism) [318]. In contrary, *ingestible electronics*, are inert devices that can be safely ingested; however, the whole system is not disposable as it can contain embedded hazards that do not come in contact with the body. There is thus a need for controlled administration and recollection after use. Edible devices are not always fully biodegradable as they often contain inert or biocompatible non‐degradable materials, such as gold for contacts [318].	No metrics for edible electronics Material candidates can be selected from food‐safe standards such as GRAS (generally recognized as safe), or EFSA (European Food Safety Authority)

### 5.2 The Role of Conjugated Polymers in Transient Bioelectronics

Early examples of transient bioelectronic devices featured partially dissolvable components, using degradable substrates while leaving insoluble electronic residues [319, 320]. While substrates typically constitute over 99% of the device's weight [321], it is crucial that every component degrades into non‐toxic, non‐bioaccumulative products. In 2012, Hwang et al. demonstrated that nanometer‐thick single‐crystal silicon dissolves in phosphate buffer solution at 37°C within 8–20 days, marking the advent of fully transient electronics [301]. Early research focused on inorganic silicon‐based transient devices but soon expanded to include a diverse range of materials such as metals, polymers, and semiconductors [322, 323, 324, 325].

In this section, we discuss strategies to develop degradable organic bioelectronics, particularly focusing on conjugated polymer core components, as well as strategies to evaluate their degradation profile. For a more comprehensive review of biodegradable materials for transient electronics, readers are referred to Feig et al. and Stephen et al. [326, 327].

Polymer degradation depends on many factors, including chemical composition, molecular structure, hydrophilicity, crystallinity, as well as environmental conditions such as temperature, humidity, and pH. Different degradation pathways exist and depend on the intrinsic properties of the initial material and degradation conditions. The main degradation mechanisms relevant for transient organic bioelectronics are hydrolytic (hydrolysis), oxidative, and enzymatic degradation. Hydrolysis occurs when chemical bonds are cleaved by the action of water molecules. The main classes of polymers that are susceptible to hydrolysis include polyesters, polyethers, polycarbonates, polyanhydrides, and polyamides [328]. The degradation rate can be affected by many factors, e.g., temperature, humidity, degree of crystallinity, polymer's polarity, hydrophilicity [328, 329].

One of the early strategies for obtaining degradable conjugated polymers involved the synthesis of copolymers composed of conjugated oligomers linked through hydrolysable bonds, an approach first exemplified by Lei et al. in 2017 (Figure 1a) [16]. For example, Rivers et al. reported a degradable polymer composed of pyrrole−thiophene−pyrrole trimers connected via ester linkages, achieving degradability with a conductivity on the order of 10^−4^ S cm^−1^ (much lower than the 1 ± 100 S cm^−1^ for non‐degradable polypyrrole) [356]. Bao et al. showed that hydrolysable linkages can be incorporated directly into semiconducting polymers to enable fully disintegrable devices. The researchers used Schiff base polycondensation to synthesize conjugated polymers with reversible imine bonds and good charge carrier mobilities (OFET hole mobility 0.34 ± 0.04 cm^2^ V^−^^1^ s^−^^1^). They then assembled the polymer with 1–2 µm thick regenerated cellulose substrate, Al_2_O_3_ dielectric layer, and iron contacts to fabricate OFETs that disintegrate under mild acidic conditions within 30 days [16]. The use of iron (work function ≈ 4.8 eV) instead of gold (≈ 5.1 eV) as the metal contact increased the energetic mismatch with the polymer HOMO level, hindering hole injection and ultimately reducing the hole mobility to 0.12 cm^2^ V^−^^1^ s^−^^1^. While this work represents one of the first examples of completely degradable OFETs, one degradation product, p‐phenylenediamine, is known to be toxic depending on exposure route and concentration (median lethal dose (LD50) for oral ingestion around 100 mg kg^−1^ in most animals) [330, 331]. This highlights the importance of thoroughly assessing the identity and quantity of degradation products, as they can pose greater toxicity risks than the original conjugated polymers, with health effects observed even at picogram levels. A comprehensive evaluation of the degradation mechanism and products as function of the degradation conditions (e.g., in vivo, soil, compost, etc.) is essential to ensure safe and sustainable transient bioelectronic devices [314, 332, 333, 334].

Oxidative degradation occurs when the material is degraded by the action of oxygen. The degradation is initiated by radical formation onto the backbone, which then reacts with O_2_ to form unstable peroxy radicals (ROO•), which eventually results in chain scission [335]. Oxidation is a slow process, which often requires external stimuli to initiate the process, e.g., light, heat, catalysts, metals, or metal ions. Huang et al. developed self‐degradable conjugated polymer nanoparticles comprising imidazole units. Under light irradiation, the conjugated polymer efficiently generates superoxide radicals (O_2_^−^·), which trigger its self‐degradation into small molecules, effectively turning off reactive oxygen species generation post‐therapy [336]. In this case, toxicity was evaluated at increased nanoparticle concentration (up to 80 µg mL^−1^), showing negligible phototoxicity of their degradation product. However, the results are limited to immortalized tumor cell lines (i.e., HeLa and 4T1), which do not fully translate the in vivo environment, particularly with respect to oxidative stress responses, immune interactions, and degradation products fate.

Another approach to develop degradable semiconductors is the use of conjugated small molecules with natural origins, such as dyes like indigo, anthraquinone, or β‐carotene [337, 338, 339, 340]. However, as discussed in Section 2.2, some of these building blocks exhibit much lower charge carrier mobilities compared to synthetic polymers. Moreover, these molecules can be highly resistant to microbial or enzyme attach due to their high stability of H‐bonding and their low solubility in water, reducing their bioavailability. Furthermore, some dyes, such as indigo, are reported to be toxic to some microbes, animals, and plants [341]. Several fungi and bacteria have shown the ability to degrade to some extent these types of compounds. However, while the degradation of indigo and indigoids, e.g., indigo carmine, tyrian purple, anthraquinone‐based dyes, have been extensively studied, other molecules, such as epindolidiones and quinacridones, have not been addressed so far [342].

Table 5 provides an overview of the published intrinsically degradable conjugated polymers along with their thin film mobilities. As of now, many degradation studies focused on abiotic degradation, with the exception of conjugated polymer nanoparticles, where in vitro and/or in vivo biodegradations were performed [337, 343, 344]. Most importantly, for most intrinsically degradable conjugated polymers, degradation is limited to backbone cleavage, with no evidence indicating whether they can be further mineralized or metabolically cleared by the body. A notable exception is poly(deca‐4,6‐diynedioic acid) (PDDA), which decomposes under sunlight and air to succinic acid and CO_2._ However, its device‐level performance and suitability for applications remain unclear [338].

**TABLE 5:** Summary of the chemical structure, degradation conditions, and thin film mobility of the main types of intrinsically degradable conjugated polymers reported in literature. We note that for all entries degradation is limited to backbone cleavage. The targeted degradable linkages are highlighted in red.

Chemical structure ^a^	Degradation conditions	Degradation form factor	Best mobility (cm^2^ V^−1^ s^−1^)	Reference
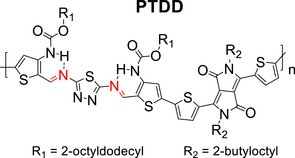	0.09 M TFA	Solution	3.8 × 10^−3^ ^a^	[345]
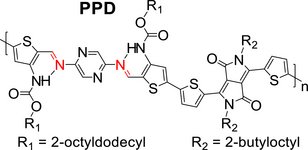			0.140 ^a^	
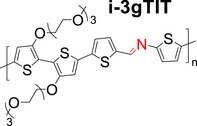	10 vol %, 0.1 M TFA	Solution	1.8 ± 0.17 ^b^	[346]
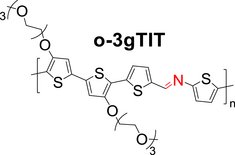			0.21 ± 0.02 ^b^	
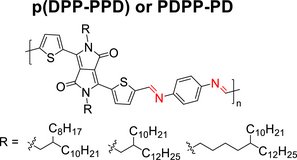	0.01 M TFA	Solution and thin film	0.34 ± 0.04 ^a^	[347]
	1 v/v % acetic acid in water			[16]
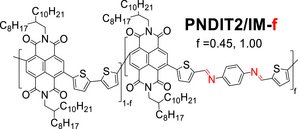	0.1 M TFA	Solution	0.04 ± 0.01 ^a^	[348]
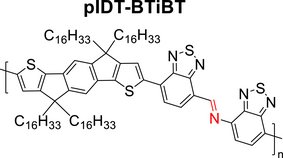	∼0.01 mg mL^−1^ TFA	Solution and thin film	0.016 ± 0.002 ^a^	[349]
**pIDT‐10BTiPh**: 10 mol% BTiPh:BTiBT			0.08 ± 0.01 ^a^	
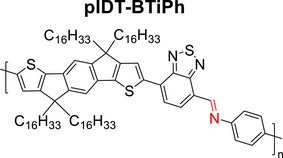			0.010 ± 0.002 ^a^	
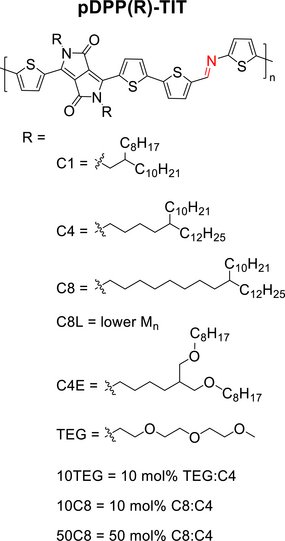	8mM to 0.5 M TFA	Solution and thin film	C1: 0.32 ± 0.05 ^a^	[349]
			C4: 0.65 ± 0.16 ^a^	
			C8: 0.56 ± 0.04 ^a^	
			C8L: 0.42 ± 0.07 ^a^	
			C4E: 0.13 ± 0.01 ^a^	
			10TEG: 0.46 ± 0.02 ^a^	
			10C8: 0.69 ± 0.10 ^a^	
			50C8: 0.57 ± 0.01 ^a^	
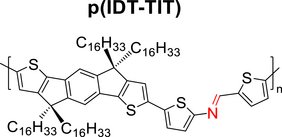	0 to 2% v/v TFA	Solution and thin film	‐ ^a^	[28]
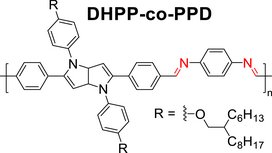	3% v/v TFA	Solution and thin film	‐ ^a^	[350]
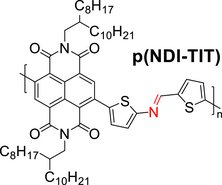	2% v/v TFA	Solution	0.102 ± 0.02 ^a^	[351]
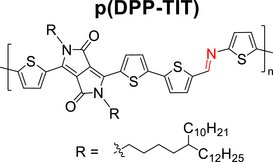			0.28 ± 0.05 ^f^	
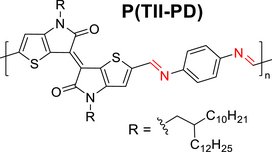	0.005 M TFA	Solution	5.8 × 10^−3^ ^f^	[352]
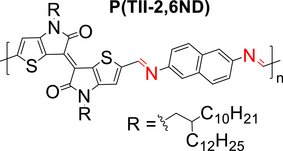			0.016 ^a^	
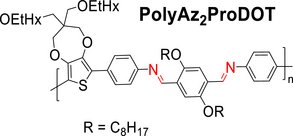	0.0067 M acid (TFA, p‐TSA, (COOH)_2_, HCl, H_2_SO_4_, H_3_PO_4_)	Solution and thin film	‐ ^f^	[353]
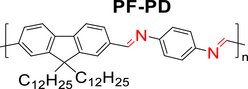	0.1% v/v TFA	Solution	‐ ^f^	[354]
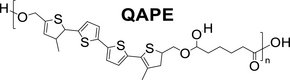	cholesterol esterase (25 units) in HEPES	Thin film	‐ ^d^	[355]
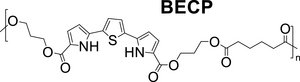	cholesterol esterase (100 units) in PBS	Thin film	Conductivity: range 10^−4^ S cm^−1^ ^d^	[356]
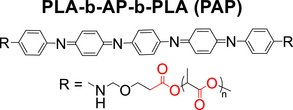	PBS 37°C pH 7.4	Thin film	Conductivity: 5 × 10^−6^ S cm^−1^ ^d^	[357]
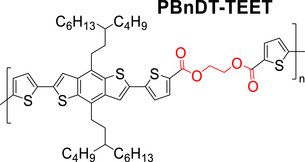	Methanolysis: THF:MeOH + K_2_CO_3_ at 50°C	Solution and thin film	‐ ^c^	[358]
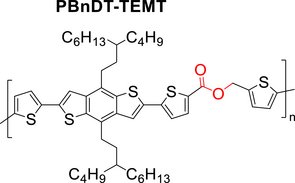			‐ ^c^	
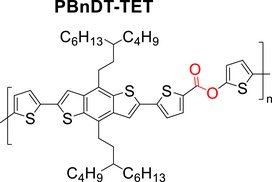			‐ ^c^	
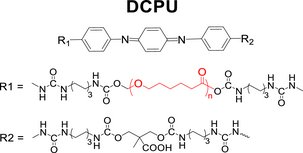	100 U mL^−1^ lipase in PBS	Thin film	Conductivity wet state: (4.7 ± 0.8) × 10^−3^ S cm^−1^ ^d^	[359]
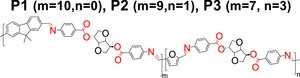	1% v/v TFA (1 M)	Solution	(2.7 ± 0.3) × 10^−2^ ^a^	[360]
			(1.6 ± 0.6) × 10^−2^ ^a^	
			(2.9 ± 0.2) × 10^−2^ ^a^	
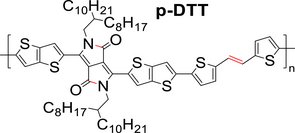	MPO (25 µg mL^−1^), H_2_O_2_ (1 mM) and NaCl (150 mM) in PBS	Nanoparticles	‐ ^e^	[350]
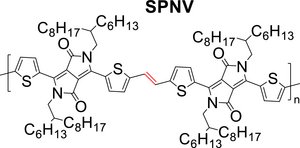	MPO (50 µg mL^−1^), H_2_O_2_ (300 µM) and NaCl (150 mM) in PBS	Nanoparticles	‐ ^e^	[337]
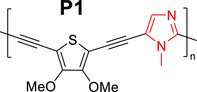	20 µM H_2_O_2_	Nanoparticles	‐ ^e^	[361]
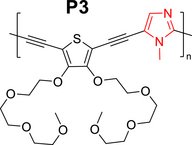			‐ ^e^	
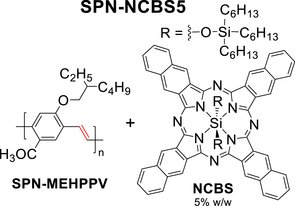	MPO (50 µg mL^−1^), H_2_O_2_ (300 µM) and NaCl (150 mM) in PBS	Nanoparticles	‐ ^e^	[362]
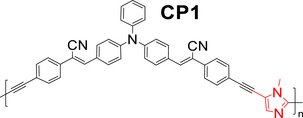	50 µM H_2_O_2_	Nanoparticles	‐ ^e^	[336]
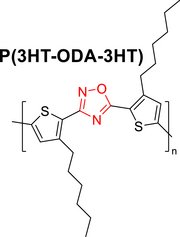	Reduction (LiAlH_4_) then HCl acid treatment	Solution	‐ ^a^	[81]
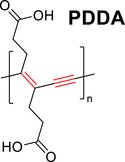	Sunlight and air	Film	‐ ^f^	[338]
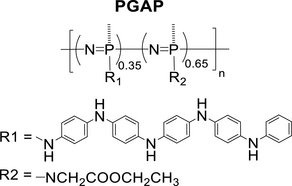	PBS 37°C pH 7.4	Thin films	Conductivity: 2 × 10^−5^ S cm^−1^ ^d^	[363]
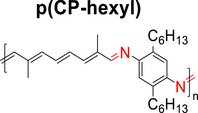	0.25 to 270 mM HCl	Solution	See ref. [364] ^a^	[365]
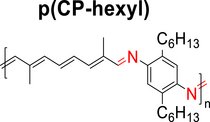	TFA (13‐130 mM) & FeCl_3_ (5‐100 µM)	Solution and thin film	(0.0054 ± 0.007) × 10^−5^ ^a^	[366]
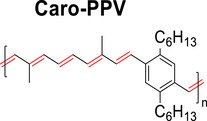			(14 ± 6.0) × 10^−5^ ^a^	

^a^ OFET;

^b^ OECT;

^c^ Solar cells;

^d^ Electroactive films;

^e^ Medical imaging, drug delivery, and/or therapeutic diagnostics;

^f^ Not stated or not tested for applications yet.

Indeed, the comparatively low mobilities of many degradable conjugated polymers highlight a major challenge in the field: the need to provide competitive charge transport while ensuring degradation into non‐toxic products after device lifetime. Stability is also an important parameter, particularly for sensors, where operational stability and shelf life are critical to ensure reliable sensing performance [339]. Future research should therefore prioritize materials that couple stable, high‐performance operation with a well‐defined and mechanistically understood degradation pathway, alongside rigorous evaluation of the identity and toxicity of the resulting degradation products. One strategy to control degradation time is to encapsulate the device with a material that degrades at a predictable rate aligned with the intended period of use [340, 367]. In this case, the degradation time is governed by chemical composition, microstructure, and thickness of the encapsulating layer [368, 369, 370]. While this approach has been widely implemented for biodegradable physical sensors, it is not applicable to biodegradable chemical sensors and biosensors, where the active sensing material must be in direct contact with the analyte‐containing fluid [371]. This is where conjugated polymers, with their synthetic tuneability, offer a distinct advantage, offering the opportunity to decouple electronic performance from degradation kinetics and thereby overcome some of the intrinsic limitations of hydrolysable metals.

Conjugated polymers with degradable linkers have been explored as a strategy for depolymerization and closed‐loop recycling. In particular, researchers have shown that ester and imine linkers enable polymers to depolymerize back to monomers, which can then be recovered and reused to resynthesize the conjugated polymer, thus achieving a chemically recyclable, closed‐loop lifecycle [352, 354, 358, 372]. While these strategies present an attractive route to achieve closed‐loop recycling for large‐area organic electronics, where substantial amounts of conjugated polymers are used, the feasibility and overall environmental impact of applying similar approaches to organic bioelectronics remain unclear. Challenges include the relatively low weight fraction of conjugated polymers compared to other device components, complex device architectures, and the presence of formulation additives and crosslinkers (as described in Section 3) that complicate separation and purification. Moreover, the overall environmental impact of recycling organic bioelectronic devices requires thorough evaluation, considering the energy and chemical inputs required for separation and depolymerization versus the impacts of device disposal or degradation without recovery. Current possibilities and associated challenges are discussed in Mantione et al. and Park et al. [372, 373]. Considerable work therefore remains to be done to broaden the range of available materials, improve material performances, and better understand the degradation mechanisms of these systems.

### 5.3 Evaluate the Degradation of Organic Bioelectronic Materials

To evaluate the degradability, compostability, resorbability, and related properties of materials, one can refer to established standards that define testing conditions, performance criteria, and certification requirements for environmental or biological breakdown. Many organizations develop standards, such as those by the International Organization for Standardization (ISO), which are highly specific depending on the materials used and their intended application. For example, ISO 22403 outlines methods to determine the aerobic biodegradability of plastics in an aqueous medium using dissolved organic carbon (DOC) analysis. Relevant standards for each defined terms are listed in Table 6 in an attempt to guide the readers in evaluating materials properties relevant for sustainability and bioelectronic applications. It is important to note that standards are subject to periodic revision, and some previously established standards may become outdated or invalid over time. In certain cases, standards are not revised but instead formally withdrawn or discontinued.

**TABLE 6:** Summary of relevant ISO standards that can be used to assess transient electronics.

ISO 10993 Biocompatible, Assessment of biodegradation products	**Biological evaluation of medical devices** Aim: Protection of humans against biological risk due to medical devices Definitions: *Biocompatibility*: ability of a medical device or material to perform with an appropriate host response in a specific application. *Absorption*: foreign material or substance passing through or begin assimilated by cells and/or tissue over time. Application specific: adverse biological response for one application might not be regarded as such in a different situation. The body response depends on the materials, the place where it is in contact, the time of usage, etc. Tests are based on in vitro, in vivo, *ex vivo* and animal model. This cannot always conclude that the same response occurs in real conditions Include a framework to plan biological evaluation rather than a rigid set test of methods that could result in unnecessary constraints or false security. Several tests are described (genotoxicity/carcinogenicity, interaction with blood, local effect after implantation, skin sensitization, toxicological risks of the constituents, etc.) as well as other information, such as sample preparation, identification and quantification of degradation products or establishment of allowable limits for leachable substances.
ISO 17556 Biodegradable	**Plastics – Determination of the ultimate aerobic biodegradability of plastic materials in soil by measuring the oxygen demand in a respirometer or the amount of carbon dioxide evolved** Definitions: *Ultimate aerobic biodegradation*: breakdown of an organic compound by microorganisms in the presence of oxygen into carbon dioxide, water and mineral salts of any other elements present (mineralization) plus new biomass. The test material is added as the unique source of carbon and energy to a flask containing soil over a period of time, and the amount of carbon dioxide produced, or oxygen consumed is measured. The degradation in percentage can be calculated by comparing the theoretical oxygen demand (or amount of carbon dioxide evolved) with the measured oxygen demand (or amount of carbon dioxide evolved). Special instruments are needed to perform such test (closed respirometer, CO_2_‐free air production system, etc.). Normal tests are typically 6 months (max 2 years). The biodegradation rate is optimized by controlling the humidity, pH and temperature (typically 25°C) of the soil. When measuring the oxygen demand, the amount of test material to use should be between 100 mg to 300 mg for 100 to 300 g of soil to outweigh background variation (typically 200 mg of material in 200 g of soil). When measuring the carbon dioxide evolution, the amount of test material should be around 2500 mg for 200 g of soil. If a final mass balance determination must be performed, higher amount of test material is needed.
ISO 14855 Biodegradable, compostable	**Determination of the ultimate aerobic biodegradability of plastic materials under controlled composting conditions – Method by analysis of evolved carbon dioxide** Definitions: *Disintegration*: physical breakdown of a material into very small fragments. The test material is placed in an inoculum (derived from compost) in an environment where the humidity, aeration, and temperature (typically 58°C) are controlled. The amount of carbon dioxide evolved is continuously monitored and the degree of material disintegration is analyzed at the end of the test. The test period should not exceed 6 months. The percentage biodegradation can be calculated with the ratio of the carbon dioxide produced to the maximum theoretical amount of carbon dioxide that can be produced from the starting material. The amount of test materials should be 50 g of dry solids in a 3 L composting vessel (the amount of material required depends on the size of the vessel).
ISO 17088 Compostable	**Plastics — Organic recycling — Specifications for compostable plastics** This standard identifies compostable plastics and compostable products made from plastics, which can be recovered by organic recycling. The compound will disintegrate and biodegrade with biowaste and produce compost as an outcome in aerobic (composting) or anaerobic (digestion) conditions followed by composting, leaving no persistent or hazardous residues. The compound must be able to (1) disintegrate during composting, (2) have an ultimate aerobic biodegradation, and (3) have no adverse effect on terrestrial organisms. The disintegration can be tested as described in ISO 16929 (the product is disintegrable if at least 90% is fragmented into less than 2 mm pieces after 84 days in controlled compositing). The ultimate biodegradation can be tested as described in ISO 14855 (preferably). If not appropriate, ISO 14851, 14852 or 17556 can be used. The ecotoxicity should be assessed with a plant growth test (ISO 11269‐2), earthworms’ survival (ISO 11268) and optionally with a Nitrification inhibition test with soil microorganisms (ISO 15685)
ISO 22403 Biodegradability in marine environment	**Assessment of the intrinsic biodegradability of materials exposed to marine inocula under mesophilic aerobic laboratory conditions** This standard only assesses the biodegradability and not the constituents or their potential ecotoxic effects. The biodegradation can be tested using one of the following marine biodegradation tests: ISO 18830, ISO 19679, ISO 22404, ASTM D6691‐17, ISO 23977‐1, and ISO 23977‐2. The requirement is that the test materials should be able to mineralize at least 90% into carbon dioxide within 2 years.
ISO 22766 Disintegrable	**Plastics – Determination of the degree of disintegration of plastic materials in marine habitats under real field conditions** This standard describes the disintegration test and not the biodegradation test. The disintegration is assessed by placing the material in a marine environment for a maximum to 3 years. At least 90% of the initial material must be fragmented into pieces smaller than 2 mm, using image analysis or a sieving procedure.

As shown in Table 6, most standards to assess material degradability require sample quantities ranging from several grams to hundreds of grams. However, conjugated polymers are typically synthesized only in small quantities during early‐stage development, often on the order of tens of milligrams. For larger batch productions, yields can increase significantly to hundreds of milligrams and often gram‐scale. Only a few examples, such as PEDOT:PSS and PBFDO, have been synthesized in formulations over a liter scale, as discussed in Section 2 [28]. These quantities significantly limit the possibility to perform standard degradation tests on many newly synthesized polymeric materials. For instance, ISO 17566 requires 200 mg per test when measuring oxygen demand and 2.5 g per test when measuring CO_2_ evolution, while ISO 14855 requires 50 g of dry solids for materials evaluation. The ISO 14855 standard has been used to certify the biodegradation of an inkjet‐printed electrochromic device [374]. While, with 79.1% degradation within 9 weeks, the device overcame the 70% degradation required to be certified as biodegradable under the ISO 14855 standard, the remaining 20% of the display did not degrade. Among the non‐degraded parts, PEDOT:PSS failed the biodegradability test according to ISO 14852.

As such, there is a need to adapt existing test methods from the field of degradable polymers to make them suitable for assessing the degradability of conjugated polymers during the development phase.

The section below describes suitable test methods that require small amounts of test material.

Mass spectrometry can be used to assess both the starting and residual materials, i.e., leachable and residues [375, 376]. Only a few nanograms to micrograms are needed per run, so the whole study can be performed with 1–2 mg. Mass spectrometry gives information about the molecular mass, structural information to determine whether the initial compound has undergone modification. Degradation products can be identified individually, and kinetic studies can also be performed. This method is very sensitive and can detect traces of products down to the low ng mL^−1^ range, depending on the instrument configuration [377]. This method enables both qualitative and quantitative analysis.

Fourier‐Transform Infrared spectroscopy (FTIR) can be used to obtain structural information about chemical bonds. FTIR detects the vibrational frequencies of chemical bonds and can therefore reveal whether bonds have been broken during degradation or if new bonds were formed (for example, carbonyl bonds appearing during oxidation or hydroxyl bonds during hydrolysis) [364, 378, 379]. This method is qualitative and can confirm the presence or absence of different functional groups, enabling the monitoring of chemical modifications during degradation. Moreover, only microliters of liquids or a few milligrams of solids are required, and thin films, requiring even less material, can be analyzed. This method is primarily used for qualitative analysis, although it can also be applied for quantitative analysis [380].

Nuclear Magnetic Resonance (^13^C and ^1^H NMR) spectroscopy provides information about the magnetic environment of nuclei, which reflects the chemical structure and connectivity of the compound, such as which atoms are bonded to which [381]. NMR can reveal structural information, such as the appearance or disappearance of functional groups within the compound. Kinetic studies can be performed to monitor the formation of degradation products in real time. NMR is both qualitative and quantitative. This method typically requires slightly larger quantities of materials (0.5 to 10 mg), although smaller amounts can be used depending on the NMR field strength. For commonly used NMR spectrometers (400–600 MHz), detection limits are typically in the micromolar range, making NMR roughly one to three orders of magnitude less sensitive than modern LC–MS methods, which routinely reach nano‑ to picomolar concentrations. This method enables both qualitative and quantitative analysis.

UV‐Vis‐NIR spectroscopy provides valuable insights into the structure of conjugated polymers and can be used to monitor the loss of conjugation and changes in electronic structure during degradation [353, 382, 383]. A decrease in absorbance at characteristic wavelengths indicates changes in polymer structure, such as disruption of π‐stacking or chain scission. Furthermore, changes in the conjugation length result in peak shifts, and new bands can appear as a result of oxidized species. Samples can be both analyzed either as thin films or in solution, and very small amounts of material are needed, i.e., only µM concentrations, which correspond to a few micrograms. This method enables both qualitative and quantitative analysis.

Differential Scanning Calorimetry (DSC) and Thermogravimetric Analysis (TGA) are used to monitor the polymer crystallinity and chain configuration [377]. These thermal methods can indicate the onset temperature of thermal degradation. They can also be used to analyze the residues from degradation, and to assess oxidative stability and thermal stability [384, 385]. Typically, 5–10 mg are required, but the amount can be reduced to 0.5–2 mg. Samples can be in solid, thin film, or liquid form. These thermal techniques can be challenging for conjugated polymers, however, as they do not show a clear glass transition [386].

Size exclusion chromatography (SEC) provides information about the molecular weight distribution and polydispersity, which can indicate, for example, changes in polymer chain length and detect backbone cleavage [387, 388]. Typically, 0.1 to 1 mg per run is required.

Raman spectroscopy can be used to obtain a molecular fingerprint of the constituent structure [389, 390, 391]. Similar to FTIR, Raman probes bond vibrations but through different vibrational modes. Structural changes upon degradation can therefore be monitored by analyzing peak intensity and their position. Thin films can also be analyzed, typically requiring only microgram‐scale samples. However, the sample must exhibit Raman‐active vibrational modes. In Raman spectroscopy, a vibration is observed when it induces a change in polarizability, whereas FTIR detects vibrations that involve a change in dipole moment.

Atomic Force microscopy (AFM) can be used to analyze the morphological changes during degradation and the local mechanical properties of the sample [392, 393]. It requires negligible amounts of material in the form of a thin film. Moreover, using conductive tips enables monitoring local changes in electrical current of the material, which could be due to degradation.

X‐ray Photon Spectroscopy (XPS) can be used to analyze thin films, as it is a surface‐sensitive method. XPS irradiates samples with soft X‐rays and measures the kinetic energy of emitted photoelectrons, providing information about the chemical composition and bonding environment. This method is often used to study processes such as photo‐oxidation [394, 395]. XPS is primarily used for qualitative analysis, although it can also be applied for quantitative analysis [396]. Each method has its advantages and limitations; therefore, a combination is needed to provide mechanistic understanding of conjugated polymers degradation.

The field of transient organic bioelectronics is still in its infancy, and its definition remains broad, encompassing not only biodegradable devices but also devices that can, among others, dissolve, disintegrate, physically disappear, or be resorbed. The amount of conjugated polymer in bioelectronic devices is, in most cases, quite small compared to insulating components, sometimes accounting only about 10^−4^% of the total device carbon [374]. Yet, as discussed in Section 2, even small material fractions can carry disproportionate embedded carbon burdens, particularly when their synthesis relies on multistep or energy‐intensive processes, indicating the need to evaluate degradability alongside the full life‐cycle carbon footprint of the device.

Evaluating toxicity is also of paramount importance, as certain degradation products might induce adverse effects even at very low doses. If these aspects are thoroughly addressed, conjugated polymers could provide a sustainable solution for temporary applications, such as environmental sensors and short‐term biomedical monitoring devices.

## 6 Conclusions and Perspectives

### 6.1 Sustainability Considerations Across the Life Cycle of Organic Bioelectronic Devices

**FIGURE 9:**
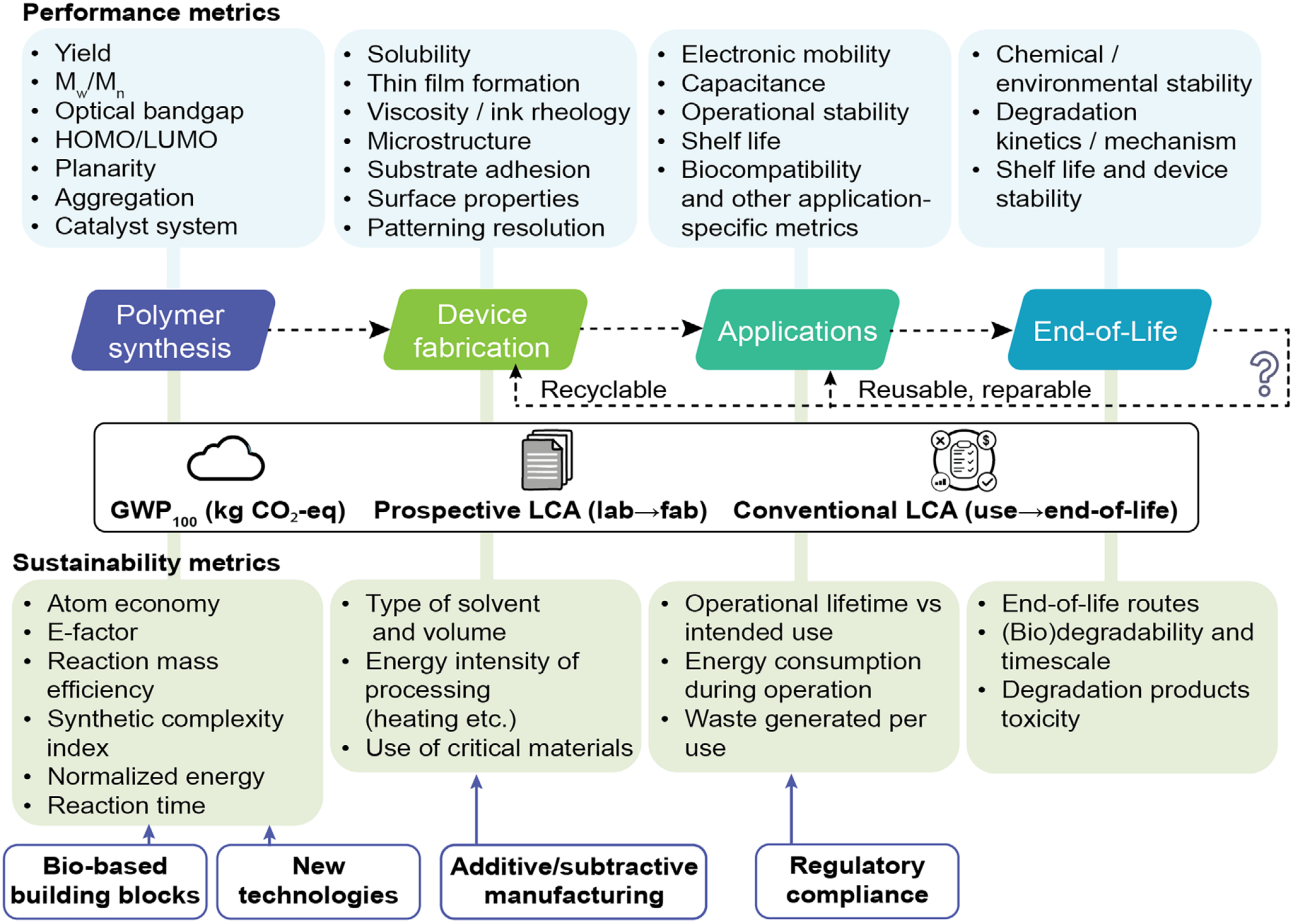
Summary of performance and sustainability metrics across the different stages of device development. Performance metrics (blue boxes) and sustainability metrics (green boxes) from material design, to fabrication, applications, and end‐of‐life considerations. In the middle are highlighted the main tools to assess sustainability across these four domains. Moreover, purple boxes highlight opportunities and impacts beyond performance and sustainability.

Building on the discussion presented throughout this work, it is essential to distill these insights into key considerations for the design of organic bioelectronic devices from a sustainability perspective (Figure 9). This section aims to address this objective.

#### 6.1.1 Core OMIEC components

The constant push toward the highest possible device performance has resulted in increased synthetic complexity for many modern conjugated polymers [397]. Increases in SC Index, as defined in section 2.1, are associated both with higher materials costs, scaling linearly with the number of synthetic steps [398], and with a reduction in one or more sustainability metrics, such as overall yield, solvent and reagent consumption, waste generation (E‐factor), and energy demand per unit of product. Reporting sustainability metrics alongside performance parameters, as exemplified by Kimpel et al. [28], is the best way to ensure that the field of organic bioelectronics moves towards materials that are both high‐performing and sustainable, with synthetic choices guided not only by application‐related metrics (e.g., conductivity and mobility) but also by quantitative, comparable measures of environmental and resource efficiency.

While significant emphasis has been placed on the development of degradable conjugated polymers, particularly for implantable devices, it is important to recall that the active layer represents only a minor fraction of the total device mass, with substrates accounting for up to 99% of the overall weight [321]. This limited contribution is further reflected at the energy level: in one of the few life cycle assessments conducted on a complete device (cradle‐to‐gate, end‐of‐life excluded), Valverde et al. reported that PEDOT accounts for only 0.16% of the total energy required for the fabrication of a multilayer organic solar cell [39]. Nevertheless, the multistep synthesis of conjugated polymers—especially heterocoupling synthetic methods with preparation of precursors—can substantially increase the environmental footprint of device fabrication, especially when additional purification steps require 2–5 L of chlorinated solvents per gram of product [399].

One strategy to decrease the amount of conjugated polymer while maintaining device performance consists in “diluting” it within an insulating matrix. This approach has been demonstrated using p(N‐T), a copolymer based on aza‐substituted bis(2‐oxoindolin‐3‐ylidene)‐benzodifuran‐dione acceptor units and thiophene donor units, blended with 10 kDa polystyrene. In this system, the insulating polymer promotes improved molecular packing, leading to an increase in charge carrier mobility by one order of magnitude with respect to the pristine conjugated polymer film (from 4.3 to 13.4 F V^−1^ cm^−1^ s^−1^) [400]. More broadly, such blending strategies offer a route to balance electronic performance with additional functionalities, including stretchability or partial degradability [347].

#### 6.1.2 Solvents

Important trade‐offs also arise in the context of thin film formation for organic bioelectronic devices. High‐quality films are commonly obtained using halogenated or aromatic solvents with high polarizability and low boiling points, which limit phase separation and promote optimal device performance. Corzo et al. notably showed that, in the field of organic solar cells, the highest‐performing devices are often associated with solvents exhibiting a median lethal dose below 4 g kg^−^^1^ such as aromatics and halogenated organic solvents [99].

Improving the sustainability of organic electronic formulations therefore requires a shift toward alternative solvents, such as alcohols, terpenes, cyrene, or other biobased aprotic solvents (Figure 3). However, maintaining high polarizability without halogenation typically necessitates bulkier solvent structures, which in turn leads to higher boiling points. This results in more demanding processing conditions and can compromise film homogeneity during device fabrication. Despite their limited processability, green solvents can be tailored through blending with low‐boiling‐point and miscible co‐solvents, ultimately enabling improved film formation and enhanced homogeneity as we discussed in Section 2.2 (Solvent considerations).

#### 6.1.3 Conductive Materials/Electrode Contacts

A more rational device design also requires careful selection of conductive materials. Gold has historically been favored for electrode fabrication; however, its extraction and refining confer a significantly higher global warming potential (GWP ≈ 4.9 × 10^4^ kg CO_2_e per kg), two orders of magnitude greater than that of Ag, Cu, or Al [27, 47]. In many cases, the chemical inertness of gold may not be critical, particularly for transient devices or encapsulated architectures where only the active channel is exposed to the biological environment. Nevertheless, mismatches between organic bioelectronic materials and electrode work functions may negatively affect device performance, as discussed in Section 5.2.

#### 6.1.4 Device Fabrication

As discussed in Section 3, fabrication processes also play a central role in determining the environmental footprint of organic bioelectronic devices. Since the first life cycle assessment (LCA) study addressing organic solar cells (OSCs) in 2011 by Espinosa et al. (Figure 1b) [401], an increasing number of LCA investigations involving organic bioelectronic materials have been reported, including the work of Teuerle et al. on device processing. These studies provide valuable guidance for selecting fabrication strategies that achieve the desired resolution while minimizing environmental impact [27, 402]. Conversely, the increasing adoption of extreme ultraviolet (EUV) patterning in the semiconductor industry may reduce the environmental footprint of photolithographic processes by eliminating the need for photoresists [403], thereby avoiding the associated use of resists and developers, which constitute the largest contribution to CO_2_ emissions in photolithography material flows (approximately 25 kg CO_2_‐eq per 100 substrates) [27].

These differences, however, must be evaluated alongside important trade‐offs. Photolithography remains unmatched in terms of device resolution, whereas printing techniques still face limitations, particularly regarding the scalability of inkjet‐based approaches. Consequently, applications requiring high‐resolution features continue to rely predominantly on photolithographic fabrication, while screen printing emerges as a more suitable alternative when scalability and manufacturing throughput outweigh constraints related to material consumption or device geometry [27].

Recent strategies combining either spin coating or inkjet printing with laser ablation have enabled the fabrication of OECTs with feature resolutions down to 2 µm without the need for conventional lithographic equipment [146, 404]. Such approaches, based on laser patterning of maskless lithography, could become increasingly standardized and adapted to a wider range of device form factors, progressively reducing the dependence on photolithography.

#### 6.1.5 Additives and Crosslinkers

Prolonged exposure of organic bioelectronic devices to aqueous environments can lead to conjugated polymer film delamination or dissolution. This issue is often circumvented with the use of crosslinkers such as 3‐glycidyloxypropyl)trimethoxysilane (GOPS), divinylsulfone (DVS), or poly(ethylene glycol)diglycidyl ether (PEDGE) that allow to create a covalent network between the substrate and conjugated polymer [255]. If these additives extend the stability over time, they introduce irreversible chemistries that might compromise either recyclability or degradability of the conjugated polymer layer. With each additive comes another layer of complexity, and therefore alternative strategies should focus on the integration of dynamic non‐covalent interactions, such as the engineering of catechol moieties inspired by bioelectronic hydrogels that could reversibly enhance the adhesion of the conjugated polymer to the substrate [405].

#### 6.1.6 System Considerations

Beyond material choice, system‐level considerations play a dominant role in the overall footprint. In particular, the integration and processing of electronic components, such as printed circuit boards (PCBs), can outweigh the impact of the sensing materials themselves. Yang et al. reported that sensing materials account for only 3% of the total GWP, whereas flexible PCB assembly contributes up to 95.9% [47]. Ion‐exchange membranes, commonly used as gatekeeping layers in bioelectronic sensing platforms and biofuel cells, also raise important sustainability concerns. In particular, the projected increase in the use of PTFE‐based membranes is problematic given the persistence and bioaccumulative nature of fluorinated materials [406]. Alternative membrane materials, including cellulose, chitosan, and poly(phenylene oxide)‐based systems, have emerged as promising candidates capable of compensating for their lower proton conductivity through higher ion‐exchange capacity [407]. Beyond material substitution, more rational device design is equally important. For instance, OECTs intended for biological recording are not required to withstand highly acidic or basic environments, thus polyethylene oxide as an ionic membrane material should be as suitable as Nafion for such applications.

This highlights the importance of developing alternative embedded systems. In this context, transistors with intrinsic amplification, such as OECTs and EGOFETs, offer a distinct advantage due to their low operating voltages, enabling the use of simple and versatile control platforms such as Arduino‐ and ESP32‐based systems, as well as modular designs (Raspberry Pi, ADS1115 ADC, etc.) [211, 408].

Costs are also critical to ensure that these technologies are eventually translated from lab to market. For instance, high‐throughput in vitro screening platforms can justify more expensive device architectures (e.g., $10–100 per device) as the costs difference between in vitro and in vivo testing is large ($1300 vs. $11 500 for a phototoxicity test, respectively) [409]. In other fields such as disposable food‐monitoring labels, cost constraints are far stricter, and typically need to remain below $0.10–1 per unit [410], corresponding to 10%–15% of the final price of the product [253].

Many LCAs adopt either a cradle‐to‐gate or cradle‐to‐gate scope, covering only the manufacturing and fabrication stage and, in some cases, the use phase, while excluding end‐of‐life scenarios. While these LCAs can be useful at a prototyping stage to prioritize aspects such as materials and fabrication choices, to draw conclusions about the true environmental impact of organic bioelectronic devices, cradle‐to‐grave assessments, encompassing material sourcing, device fabrication, operational lifetime, and end‐of‐life management, are critically needed [27].

#### 6.1.7 Reusability, Recyclability, and (Bio)Degradation

Over the past decade, there has been a rise in research focus on degradable materials and devices. While degradation can be advantageous for certain applications, such as embedded systems where components are difficult to disassemble and recycle or transient implantable devices to avoid secondary surgeries for device extraction, it does not necessarily translate into improved environmental performance. For applications where recycling practices exist, such as e‐textiles [185], degradation might instead promote downcycling, resulting in a loss of valuable materials. In these cases, designing for reusability or recyclability might offer more robust pathways toward circular material flows.

It is important to note that each application requires a distinct transience timescale to remain functionally relevant. This can range from one week for fresh meat to several months for pathogen detection in crop fields [267, 411]. Achieving this objective requires careful design not only of the substrate, but also of the encapsulation layer, as these components may need different degradation timelines to ensure proper device operation. Transient substrates and encapsulation materials are comprehensively reviewed by Ding et al. [412].

### 6.2 From Implementing Sustainable Metrics to Decision‐Making

Informed decision‐making toward more sustainable organic bioelectronic materials and devices ultimately requires a clearer understanding of the environmental footprint associated with the various materials and fabrication processes. The establishment of harmonized sustainability metrics, spanning material selection and device processing (e.g., GWP, toxicity), is therefore essential to move toward a holistic framework for sustainable electronics.

Achieving such a framework, however, depends on overcoming key challenges including the lack of data, expertise, and drive towards integrating sustainability metrics.

- **Lack of data**. Comprehensive datasets covering both established and emerging processes remain largely unavailable. While green chemistry principles provide valuable guidance at the molecular scale, through metrics such as atom economy and E‐factor, extending these considerations to device fabrication requires broader data collection, including solvent usage during purification and cleaning, as well as processing conditions such as temperature and time. This calls for closer interdisciplinary collaboration between life cycle analysts, organic chemists, and materials scientists to generate actionable datasets and guide more sustainable practices, or complete preexisting datasets such as the Microelectronics LCA database [413]. In this regard, the emergence of high‐throughput and flow chemistry approaches offer a promising route to quantify and optimize synthetic metrics with greater precision.
- **Lack of expertise**. Although interest in LCA is steadily increasing, most research groups in chemistry and materials science still lack the expertise required to perform comprehensive sustainability analyses, thereby limiting the depth of sustainability‐oriented research. Establishing a holistic understanding of a device's environmental footprint requires scientists to consider not only conventional synthetic metrics, such as yield and performance, but also frequently overlooked parameters that strongly influence the overall impact, including energy consumption and solvent usage, and also the raw material impacts. Lucas et al. demonstrated that this last metric account to 70% of the total LCA contribution when it comes to chemistry [414, 415]. In this regard, chemical suppliers could provide raw‐material environmental impact within the material specifications, thereby providing chemists with clearer sustainability guidance from the earliest stages of their research. Performing comprehensive risk assessments for each reaction also contributes to the accumulation of community‐wide data on exposure and toxicity, thereby supporting the improved implementation of sustainability and safety metrics. Furthermore, compiling comprehensive life cycle inventories in chemistry and materials science is often highly time‐ and data‐intensive. As a result, sustainability‐oriented research may benefit from longer evaluation timelines and publication practices that place greater emphasis on methodological depth and environmental reporting rather than rapid publication output alone.
- **Drive towards integration of sustainability metrics**. At present, only a limited number of studies report sustainability metrics alongside device performance, with notable exceptions such as the work of Kimpel et al. [75] This lack of integrated analysis makes it difficult to identify truly sustainable pathways for conjugated polymer synthesis. Moreover, studies focusing on high device performance often rely on non‐sustainable fabrication methods without discussing their environmental implications or alignment with the intended application. We also observe that the more holistic the view of device fabrication becomes, the clearer it is to identify potential mitigation strategies. For example, Yang et al. analysis of continuous glucose monitoring devices shows that the limited 14‐day lifetime of enzymatic components and the reliance on non‐renewable energy sources throughout the material flow and fabrication processes are two critical aspects of device development [47].

Addressing the sustainability gap requires a collective effort across fields. Journal editors play a key role in valuing not only performance metrics, but also the practical and environmental relevance of reported technologies. Reviewers can further encourage this shift by critically assessing materials and process choices considering sustainability. Asking, for example, to report sustainability metrics and the type of solvents used during processing alongside performance metrics.

### 6.3 The Next Generation of Sustainable Scientists in Organic Bioelectronics

Organic bioelectronics continues to attract significant interest from the research community, offering potential across a broad range of applications, as highlighted in Section 4. Two key advantages are the possibility of mass‐producing devices using solution‐processable inks and their seamless integration with living organisms, enabling high spatial resolution measurements. While the performance of conjugated polymers keeps improving, especially with the recent development of stable n‐type conjugated polymers, this momentum must not contribute further to the ongoing ecological crisis.

In 2025, a Nobel Symposium on Chemistry for Sustainability was held in Stockholm to discuss new approaches necessary to tackle the ecological crisis, primarily in response to the issues associated with polyfluoroalkyl substances [416]. In this article, we extend the ambition of this symposium, aligned with the and the EU's safe and sustainable by design framework for chemicals and materials [417], and the Sweden's Wallenberg Initiative on Materials Science for Sustainability (WISE) [418], to the field of organic bioelectronics, aiming to provide a foundational framework for researchers to advance their work in alignment with sustainability principles.

Starting with the sourcing and synthesis of conjugated polymers, we highlighted the development of new solvents, monomers, and synthetic processes designed to produce these polymers more sustainably, offering alternatives to toxic solvents or metal catalysts. While these strategies represent meaningful progress, further work is needed to fully match the molecular weight, electronic performance, or structural control achieved with established conjugated polymers [86]. We then discussed device fabrication methods, covering sustainable alternatives for material formulations and additives as well as methods that conserve energy and reduce materials waste. Following this, we presented applications where organic bioelectronics can make a significant impact, highlighting both the state‐of‐the‐art research and the current limitations that hinder widespread adoption and challenge the long‐term sustainability of these materials. Finally, we addressed end‐of‐life considerations, with a particular focus on conjugated polymers, summarizing current research and perspectives on materials and devices designed with degradation or disposal pathways.

This perspective aims to reinforce key definitions needed to establish a shared language around end‐of‐life within the organic bioelectronics’ community. Training a new generation of sustainable chemists, engineers, and material scientists will require rigorous accounting of material usage (employing sustainability metrics, as highlighted across the different sections) and careful comparison of methods based on their energy demands. In practice, this means reporting appropriate sustainability metrics together with device performance (for example, E‐factor, solvent hazard or GWP alongside mobility, transconductance, or sensitivity), assessing full device stacks rather than active layers in isolation, and explicitly stating system boundaries for any LCA‐derived claims. Progress toward more sustainable devices should also mark a shift away from the unchecked pursuit of ever‐higher performance; instead, the performance required for each application should define the limits of molecular and device engineering in a solution‐based society. At the device level, future work should prioritize avoiding PFAS‐based materials, reducing reliance on noble metals and energy‐intensive patterning, designing architectures for disassembly and reuse wherever possible, and, for transient and bioresorbable systems, matching degradation time scales and degradation products to the intended application lifetime and environment.

## Author Contributions

**Andrea Bionda**: writing – original draft, visualization, writing – review and editing, investigation. **Gwennaël Dufil**: writing – review and editing, writing – original draft, visualization, conceptualization, project administration, investigation. **Erica Zeglio**: conceptualization, funding acquisition, writing – original draft, visualization, writing – review and editing, project administration, resources, supervision, investigation. **Marine Batista**: writing – review and editing, writing – original draft, visualization, investigation.

## Conflicts of Interest

The authors declare no conflicts of interest.

## Data Availability

Data sharing not applicable to this article as no datasets were generated or analysed during the current study.
